# Health-related quality of life, psychological distress, and fatigue in metastatic castration-resistant prostate cancer patients treated with radium-223 therapy

**DOI:** 10.1038/s41391-022-00569-8

**Published:** 2022-07-08

**Authors:** Maarten J. van der Doelen, Irma M. Oving, Dirk N. J. Wyndaele, Jean-Paul van Basten, Frederiek Terheggen, Addy C. M. van de Luijtgaarden, Wim. J. G. Oyen, W. Dick van Schelven, Franchette van den Berkmortel, Niven Mehra, Marcel J. R. Janssen, Judith B. Prins, Winald R. Gerritsen, José A. E. Custers, Inge M. van Oort

**Affiliations:** 1 Department of Medical Oncology, Radboud University Medical Center, Nijmegen, The Netherlands; 2 Department of Urology, Radboud University Medical Center, Nijmegen, The Netherlands; 3 Department of Medical Oncology, Ziekenhuisgroep Twente, Almelo, Netherlands; 4 Department of Nuclear Medicine, Catharina Hospital, Eindhoven, The Netherlands; 5 Department of Urology, Canisius-Wilhelmina Hospital, Nijmegen, The Netherlands; 6 Department of Medical Oncology, Bravis Hospital, Roosendaal, The Netherlands; 7 Department of Medical Oncology, Reinier de Graaf Gasthuis and Reinier Haga Prostate Cancer Center, Delft, The Netherlands; 8 Department of Radiology and Nuclear Medicine, Rijnstate Hospital, Arnhem, The Netherlands; 9 Department of Biomedical Sciences, Humanitas University, Milan, Italy; 10 Department of Nuclear Medicine, Humanitas Clinical and Research Center, Milan, Italy; 11 Department of Nuclear Medicine, Medical Center Leeuwarden, Leeuwarden, The Netherlands; 12 Department of Medical Oncology, Zuyderland Medical Center, Sittard, The Netherlands; 13 Department of Radiology and Nuclear Medicine, Radboud University Medical Center, Nijmegen, The Netherlands; 14 Department of Medical Psychology, Radboud Institute for Health Sciences, Radboud University Medical Center, Nijmegen, The Netherlands

**Keywords:** Prostate cancer, Outcomes research

## Abstract

**Background:**

Radium-223 is a registered treatment option for symptomatic bone metastatic castration-resistant prostate cancer (mCRPC). Aim of this multicenter, prospective observational cohort study was to evaluate health-related quality of life (HR-QoL), psychological distress and fatigue in mCRPC patients treated with radium-223.

**Methods:**

Primary endpoint was cancer-specific and bone metastases-related HR-QoL, as measured by the EORTC QLQ-C30 and BM-22 questionnaires. Secondary endpoints were psychological distress and fatigue, evaluated by the HADS and CIS-Fatigue questionnaires. Outcomes were analyzed for the total cohort and between subgroups (1-3 versus 4-5 versus 6 radium-223 injections). A trajectory analysis was performed to explore HR-QoL patterns over time.

**Results:**

In total, 122 patients were included for analysis. Baseline HR-QoL, pain intensity, psychological distress and fatigue were worse in patients who did not complete radium-223 therapy. In patients who completed therapy, stabilization of HR-QoL was perceived and psychological distress and fatigue remained stable, whereas clinically meaningful and statistically significant deterioration of HR-QoL, psychological distress and fatigue over time was observed in patients who discontinued radium-223 therapy. Trajectory analysis revealed that HR-QoL deterioration over time was more likely in patients with baseline opioid use, low hemoglobin and high alkaline phosphatase levels.

**Conclusions:**

Patients who discontinued radium-223 therapy showed worse HR-QoL, psychological distress and fatigue at baseline and more frequent deterioration of HR-QoL, psychological distress and fatigue over time when compared to patients who completed therapy. Specific attention with regard to HR-QoL during follow-up is indicated in patients with opioid use, low hemoglobin and high alkaline phosphatase levels before radium-223 therapy initiation.

**Clinical trial registration number:**

NCT04995614.

## Introduction

Bone metastases of prostate cancer may cause considerable pain, impaired mobility, pathological fractures, and spinal cord compression [1]. These complications affect health-related quality of life (HR-QoL), diminish patients’ functional capacities, and lead to decreased overall survival (OS) [2–5]. In addition, androgen deprivation therapy, the standard of care for patients with metastatic prostate cancer, is known to have profound effects on physical and emotional well-being in this population, including fatigue and psychological distress [6, 7]. Furthermore, patients’ concerns about the effectivity of systemic therapies can aggravate psychological distress, and thereby negatively influences physical and mental well-being [8].

Radium-223 is a registered treatment option for patients with symptomatic bone metastatic castration-resistant prostate cancer (mCRPC). Previously, the phase 3 ALSYMPCA trial demonstrated that radium-223 improved OS and prolonged the times to first symptomatic skeletal-related event (SRE) and opioid use, irrespective of prior docetaxel chemotherapy [9–11]. Subsequent analysis revealed that a significantly higher percentage of patients receiving radium-223 experienced meaningful HR-QoL improvement when compared to patients treated with placebo [12]. Prior phase 1-2 trials had already reported pain relief after radium-223 therapy [13, 14]. Subsequently, several real-world studies have confirmed the observed decrease in pain levels during radium-223 therapy [15–17].

However, studies evaluating HR-QoL, psychological distress, and fatigue in mCRPC patients treated with radium-223 in daily practice are lacking. This is of particular importance since there is discrepancy in the observed HR-QoL between highly selected trial populations and patients in real-world practice [5, 18]. Moreover, phase 3 trials commonly used HR-QoL instruments that are not specifically designed for mCRPC patients and therefore, specific symptoms might not have been addressed in these trials [19, 20]. Additionally, the ALSYMPCA trial was conducted in the era prior to the registration of abiraterone, enzalutamide, and cabazitaxel as life-prolonging therapies for mCRPC. Therefore, the aim of the current study was to evaluate cancer-specific and bone metastases-related HR-QoL, psychological distress, and fatigue in mCRPC patients before, during and after treatment with radium-223 in daily practice. We hypothesized that there would be different HR-QoL trajectories between patients who were able to complete six injections of radium-223 therapy and patients who discontinued radium-223 therapy. Furthermore, we aimed to identify variables related to HR-QoL deterioration during the course of treatment, as patients with HR-QoL deterioration may need specific attention during follow-up.

## Materials and methods

### Study design and population

This prospective observational cohort study included mCRPC patients treated with radium-223 at eleven institutions throughout The Netherlands between April 2017 and July 2020. Eligible patients had histologically proven mCRPC with symptomatic bone metastases, and no visceral metastases. Symptomatic disease was defined as regular use of analgesics for cancer-related bone pain and/or experiencing disease-related limitations in the performance of daily activities. The initiation of radium-223 therapy was at the physician’s discretion, but the recommendations of the European medicines Agency were followed. Concomitant other anticancer treatments were not allowed except for luteinizing hormone-releasing hormone agonists or antagonists. No restrictions were applied with regard to the number of bone metastases, the PSA level, or the Eastern Cooperative Oncology Group (ECOG) performance status at baseline. Written informed consent was obtained from all patients before the start of therapy. The study was conducted in accordance with the principles of Good Clinical Practice and the Declaration of Helsinki. The study protocol was approved by the medical ethics committee (CMO 2017-3220) and the institutional review boards of all participating centers (NCT04995614).

### Study procedures

Patients were treated with intravenous injections of radium-223 (55 kBq/kg body weight) every four weeks, for maximally six doses. Consenting patients completed five questionnaires at baseline (time point T0): the European Organization for Research and Treatment of Cancer (EORTC) core QoL questionnaire (QLQ-C30) and the bone metastases module (BM-22), the Brief Pain Inventory Short Form (BPI-SF), and after a study protocol amendment in January 2018, the Hospital Anxiety and Depression Scale (HADS), and the Checklist Individual Strength–Fatigue subscale (CIS-Fatigue). The questionnaires were repeated four weeks after the third injection (time point T1) and four weeks after the sixth injection (time point T2; Supplementary Fig. 1A). In case of discontinuation of therapy, patients were asked to complete an end-of-therapy questionnaire (T1 or T2) four weeks after the last received injection (Supplementary Fig. 1B, C). Patients’ sociodemographic and clinical information were retrieved from the medical records. OS was defined as the time between the first radium-223 injection and either date of death or last follow-up date. All patients were followed until death or April 1, 2021. SREs were defined as surgery or radiotherapy to the bone, spinal cord compression, and symptomatologic pathological fractures [21].

### Study outcomes

Primary endpoint was cancer-specific and bone metastases-related HR-QoL, measured with the EORTC QLQ-C30 and BM-22 questionnaires. The QLQ-C30 questionnaire contains five multi-item functional scales (physical, role, emotional, cognitive, and social functioning), nine symptom scales, and a two-item global health status scale [22]. The BM-22 questionnaire consists of four scales assessing painful sites, pain characteristics, functional interference, and psychosocial aspects [23, 24]. All items are rated on a 4-point Likert-type response scale of 1 (“not at all”) to 4 (“very much”), with exception of the global health status scale items, which are rated from 1 to 7. Each subscale was linearly transformed to a 0–100 scale, according to EORTC scoring manuals [24, 25]. Clinically relevant changes (CRCs) in EORTC scores were defined as small (5–10 points), moderate (10–20 points), or large (>20 points) [26].

Secondary endpoints were the intensity and location of bone pain assessed by the BPI-SF, psychological distress evaluated by the HADS, and fatigue measured with the CIS-Fatigue. The BPI-SF assesses severity of pain, impact of pain on daily function, location of pain (using diagrams), pain medication, and amount of pain relief in the past week [27]. The pain severity items are rated on 0–10 scales, with 0 indicating “no pain” and 10 indicating “worst possible pain” [27]. In this study, pain diagrams and BPI-SF pain severity items “worst” (item 3) and “average” (item 5) were used to represent pain intensity at baseline. The HADS and CIS-Fatigue questionnaires were added in January 2018 after consultation with the medical psychology department of the Radboud University Medical Center for advice on the evaluation of psychological distress and fatigue. Changes over time in HADS and CIS-Fatigue scores were analyzed. The HADS contains a 7-item anxiety and a 7-item depression subscale. All items are scored on a 4-point Likert-scale ranging from scores 0 (“never”) to 3 (“almost always”). A total score of ≥11 indicates psychological distress [28]. The CRC in HADS subscale scores were defined as 1.5 points change, and 3 points change for the total HADS score [29]. The CIS-Fatigue contains eight items, and each item is scored on a 7-point Likert scale ranging from “Yes, that is true” to “No, that is not true” [30]. A score of ≥35 indicates severe feelings of fatigue [31].

### HR-QoL trajectory analysis

To explore HR-QoL patterns during radium-223 therapy, a trajectory analysis was performed. In this analysis, individual responses are classified based upon similar patterns in the outcomes of interest. For this HR-QoL trajectory analysis, we calculated the EORTC QLQ-C30 summary score, which encompasses all QLQ-C30 scales, with the exception of the financial impact and global quality of life scales [32, 33]. Summary scores were prespecified by the research team based on clinical expertise and classified as <60 (low), 60–80 (intermediate), and >80 (high) based on the cut-off for a large CRC in EORTC scores [26]. Changes in summary scores over time were classified as deteriorated (deterioration in class), stable low (low at all time points), stable intermediate (intermediate at all time points), stable high (high at all time points), improved (improvement in class), and fluctuating (varying between low, intermediate and high classes). The following baseline variables were evaluated as potential predictive factors for distinguishing between trajectory classes: age, marital state/partnership, ECOG performance status, opioid use, the number of prior therapies and hemoglobin, alkaline phosphatase (ALP) and prostate-specific antigen (PSA) levels. To verify the prognostic value of the HR-QoL classification, the OS was compared between the different baseline summary score classes and HR-QoL trajectory classes.

### Data analysis

The outcomes were analyzed for the total cohort and between prespecified subgroups, based on the number of received radium-223 injections (1–3 versus 4–5 versus 6 injections). To compare subgroups, the Chi-square or Fisher's Exact and Mann-Whitney or Kruskal-Wallis tests were used for categorical variables and nonparametric continuous variables, respectively. The paired *T*-test was used to compare patient-reported outcomes over time. OS was analyzed with Kaplan-Meier curves and stratified with log-rank tests. Univariate multinominal logistic regression analysis was used to analyze the relationship between trajectory classes and baseline variables, with odds ratios describing the probability for class membership in comparison with the reference class. ALP and PSA levels were log-transformed because of distribution skewness. Two-sided statistical analysis with *P* values <0.05 were considered to be statistically significant. Statistical analyses were performed using SPSS 25.0 (IBM, Armonk, NY, USA).

## Results

### Response to questionnaires

**Fig. 1:**
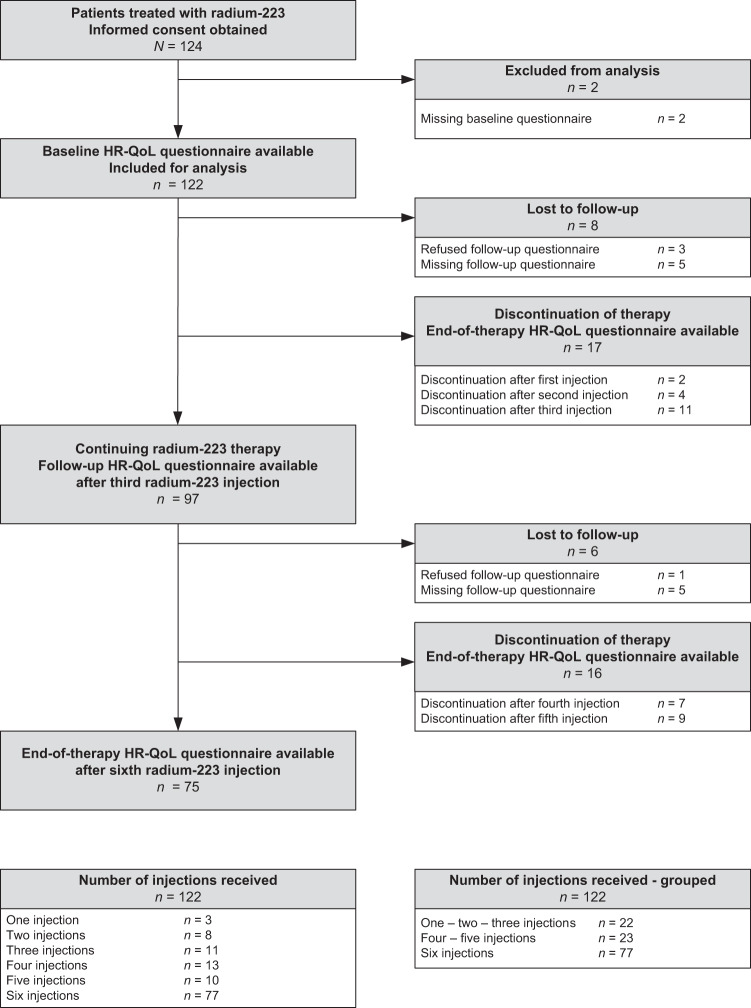
Consort diagram of patient enrollment. Enrollment based on completion of the European Organization for Research and Treatment of Cancer (EORTC) core QoL questionnaire (QLQ-C30) and the bone metastases module (BM-22).

In total, 124 patients were enrolled in this study, of whom 122 patients completed the baseline HR-QoL questionnaires and were included for analysis. A total of 327 EORTC QLQ-C30 plus BM-22 questionnaires were completed. Ninety-one (75%) patients completed all HR-QoL questionnaires, whereas 23 (19%) patients completed two HR-QoL questionnaires and eight (7%) patients completed only the baseline HR-QoL questionnaire (Fig. 1). Less participants completed the HADS and CIS-Fatigue questionnaires due to the later addition of these questionnaires to the study protocol (Supplementary Fig. 2). However, compliance rates for all questionnaires at the different time points were comparable (Supplementary Table 1). Compliance to the questionnaires decreased with each subsequent assessment due to treatment discontinuation (*n* = 17; 14%), refusal to complete follow-up questionnaires (*n* = 4; 3%), non-response to follow-up questionnaires (*n* = 3; 3%), or death (*n* = 7; 6%).

### Patient and treatment characteristics

Patient characteristics are shown in Table 1. Median age of the patients was 73 years and the majority (85%) had a good performance status (ECOG 0–1) and no or modest comorbidity (86%). Ninety-seven (80%) patients received prior abiraterone or enzalutamide. Seventy-four (61%) patients underwent prior taxane-based chemotherapy, either upfront in hormone-sensitive state (*n* = 35; 29%), in castration-resistant (*n* = 37; 30%) state, or both (*n* = 2; 2%). At time of radium-223 initiation, opioids were used in 40 (33%) patients.

**Table 1:** Baseline patient demographics and clinical characteristics.

	All patients (*N* = 122)	
Age, years, median (IQR)	73	(65–78)
Gleason score ≥8, *n* (%)	80	(65.6)
Time from mCRPC to radium-223, months, median (IQR)	21.4	(10.4–41.6)
Extent of disease, *n* (%)		
Low volume (<6 bone metastases)	15	(12.3)
Intermediate volume (6–20 bone metastases)	33	(27.0)
High volume (>20 bone metastases)	62	(50.8)
Superscan	12	(9.8)
Lymph node metastases ≥15 mm, *n* (%)	17	(13.9)
Visceral metastases, *n* (%)	3	(2.5)
Prior systemic therapies		
Number of prior registered therapies, median (range)	2	(0-5)
None, *n* (%)	12	(9.8)
Docetaxel, *n* (%)^a^	74	(60.7)
Cabazitaxel, *n* (%)	18	(14.8)
Abiraterone, *n* (%)^b^	53	(43.4)
Enzalutamide, *n* (%)	58	(47.5)
Both Abiraterone and Enzalutamide, *n* (%)	14	(11.5)
Prior skeletal-related event, *n* (%)	55	(45.1)
Opioid use, *n* (%)	40	(32.8)
ECOG performance status, *n* (%)		
ECOG 0	51	(41.8)
ECOG 1	53	(43.4)
ECOG 2-3	18	(14.8)
Hemoglobin, mmol/L, median (IQR)	7.8	(7.3-8.3)
Platelet count, ×10^9^/L, median (IQR)	240	(198-286)
PSA, ng/ml, median (IQR)	88	(29-256)
ALP, U/L, median (IQR)	142	(102-234)
LDH, U/L, median (IQR)	234	(204-280)
Concomitant bone protective agent use, *n* (%)	82	(67.2)
Bisphosphonates	25	(20.5)
Denosumab	57	(46.7)
Charlson comorbidity index, %		
6–7	105	(86.1)
8–9	15	(12.3)
≥10	2	(1.6)
Marital status, *n* (%*)		
Married/living together	105	(86.8)
Single/not living together	4	(3.3)
Divorced	3	(2.5)
Widowed	9	(7.4)
Unknown	1	
Having children, *n* (%^c^)		
Yes	99	(89.2)
No	12	(10.8)
Unknown	11	
Current profession, *n* (%^c^)		
Employed	18	(18.2)
Incapacitated	12	(12.1)
Retired/early retired	69	(69.7)
Unknown	23	

*ALP* alkaline phosphatase, *mCRPC* metastatic castration-resistant prostate cancer, *ECOG* Eastern Cooperative Oncology Group, *IQR* interquartile range, *LDH* lactate dehydrogenase, *PSA* prostate-specific antigen.

Superscan refers to a bone scan showing diffuse, intense skeletal uptake of the tracer without renal and background activity.

^a^Including 37 (30.3%) patients who were treated with upfront docetaxel for metastatic hormone-naïve prostate cancer.

^b^Including 2 (1.6%) patients who were treated with upfront abiraterone for metastatic hormone-naïve prostate cancer.

^c^In case of missing data, valid percentages were calculated.

Seventy-seven (63%) patients completed radium-223 therapy. Twenty-two (18%) and 23 (19%) patients received 1–3 and 4–5 injections, respectively. At the moment of survival analysis, 79% of the patients had died and the median OS was 12.8 months (95% CI 11.1–14.4; Supplementary Fig. 3A).

### Baseline HR-QoL, pain intensity, psychological distress, and fatigue

Baseline HR-QoL, pain intensity, psychological distress, and fatigue scores are presented in Table 2. Physical and role functioning were affected most, with mean scores of 71.5 and 64.3, respectively. Most reported symptoms were pain, fatigue, and insomnia. At baseline, 47% of the patients had psychological distress and 50 (52%) patients reported severe feelings of fatigue. Mean total HADS and CIS-Fatigue scores at baseline were 11.0 and 33.5, respectively, indicating moderate to high levels of psychological distress and fatigue among the patients.

**Table 2:** A. Baseline patient-reported outcomes on cancer-specific and bone metastases-related health-related quality of life and bone pain intensity. B. Baseline patient-reported outcomes on psychological distress and fatigue.

A								
	**Total cohort (*N*** = **122)** Mean (SD)		**Subgroup 1-3 injections (*N*** = **22)** Mean (SD)		**Subgroup 4-5 injections (*N*** = **23)** Mean (SD)		**Subgroup 6 injections (*N*** = **77)** Mean (SD)	
QLQ-30 functional scales^a^								
Global health status	62.5	(20.4)	54.9	(27.8)	58.3	(21.9)	65.9	(16.6)
Physical functioning	71.5	(22.5)	63.6	(24.6)	72.2	(21.7)	73.5	(22.0)
Role functioning	64.3	(30.9)	58.3	(33.2)	62.3	(34.5)	66.7	(29.2)
Emotional functioning	77.9	(21.3)	76.9	(20.4)	73.6	(25.8)	79.5	(20.2)
Cognitive functioning	83.5	(19.7)	80.3	(22.8)	76.1	(22.9)	86.6	(17.1)
Social functioning	79.5	(25.0)	75.8	(33.2)	77.5	(26.9)	81.2	(21.7)
QLQ-30 symptom scales^b^								
Fatigue	36.0	(23.5)	43.4	(28.5)	34.3	(23.7)	34.3	(21.7)
Nausea/vomiting	7.5	(16.7)	7.6	(13.3)	13.8	(25.5)	5.6	(13.9)
Pain	37.8	(29.8)	51.5	(35.2)	38.4	(29.1)	33.8	(27.6)
Dyspnea	17.2	(25.8)	25.8	(29.0)	15.9	(24.3)	15.2	(25.1)
Insomnia	19.9	(24.5)	21.2	(24.2)	18.8	(22.1)	19.9	(25.5)
Appetite loss	14.8	(26.8)	19.7	(30.3)	18.8	(33.1)	12.1	(23.5)
Constipation	10.1	(20.5)	9.1	(23.4)	4.3	(11.5)	12.1	(21.6)
Diarrhea	8.2	(23.2)	4.5	(11.7)	5.8	(21.7)	10.0	(26.0)
Financial difficulties	7.2	(17.3)	4.5	(11.7)	8.7	(18.0)	7.5	(18.5)
BM-22 symptom scales^b^								
Painful sites	24.9	(16.6)	30.0	(19.7)	23.2	(16.8)	24.0	(15.5)
Pain characteristics	29.2	(24.1)	39.9	(27.6)	27.1	(22.9)	26.8	(22.9)
BM-22 functional scales^a^								
Functional interference	74.1	(20.7)	68.6	(24.0)	75.5	(19.9)	75.2	(20.0)
Psychosocial aspects	60.5	(18.2)	56.6	(21.8)	59.5	(21.1)	61.9	(16.1)
BPI-SF								
Average pain score	3.4	(2.7)	4.2	(2.9)	3.3	(2.8)	3.1	(2.6)
Worst pain score	4.4	(3.2)	5.3	(3.3)	4.1	(3.1)	4.2	(3.2)
B								
	**Total cohort (*N*** = **96)** Mean (SD)		**Subgroup 1-3 injections (*N*** = **19)** Mean (SD)		**Subgroup 4-5 injections (*N*** = **18)** Mean (SD)		**Subgroup 6 injections (*N*** = **59)** Mean (SD)	
HADS^c^								
Anxiety score	5.5	(4.0)	6.0	(4.2)	6.2	(3.2)	5.2	(4.1)
Depression score	5.5	(4.3)	7.1	(5.7)	5.9	(3.9)	4.8	(3.7)
Total score	11.0	(7.8)	13.1	(9.4)	12.1	(6.5)	10.0	(7.5)
CIS Fatigue^c^								
Fatigue severity subscale	33.5	(13.6)	33.5	(16.0)	36.0	(11.5)	32.7	(13.5)

Mean health-related quality of life scores range from 0 to 100.

^a^Functional and global scales: high scores indicate high level of functioning.

^b^Symptom scales: high scores indicate high symptom burden.

^c^High scores indicate high symptom burden.

Baseline HR-QoL, pain intensity, psychological distress, and fatigue were worse in patients who did not complete radium-223 therapy. When compared to patients who completed radium-223 therapy, patients who received 1-3 radium-223 injections had clinically relevant lower baseline global health status and physical functioning scores. In addition, these patients showed higher baseline pain and dyspnea scores when compared to the other patients. The sacroiliac region was the most prevalent (35%) reported location of pain (Supplementary Fig. 4).

### Changes in HR-QoL, psychological distress, and fatigue over time

**Fig. 2:**
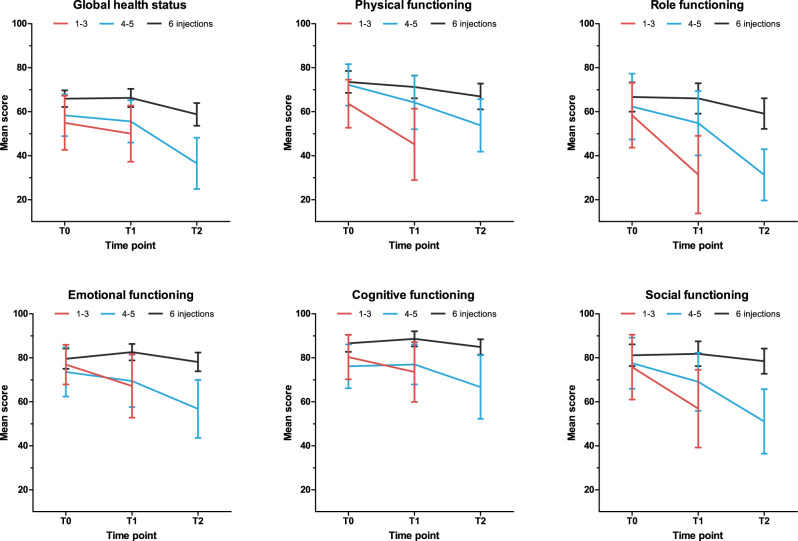
Course of cancer-specific health-related quality of life (EORTC QLQ-C30 functional scales and global health status) over time in patients treated with radium-223 for metastatic castration-resistant prostate cancer. Bars indicate mean scores with 95% confidence intervals.

Patients who completed radium-223 therapy experienced stabilization of cancer-specific HR-QoL, with only small changes in the global health status and physical and role functioning by end of treatment (Fig. 2). Moderate CRCs were observed in the EORTC QLQ-C30 domains fatigue, nausea/vomiting, and appetite loss in this subgroup (Supplementary Table 2). No increase in psychological distress was found in these patients.

In contrast, in patients who discontinued radium-223 therapy, clinically meaningful deterioration on all EORTC QLQ-C30 functioning scales was observed (Supplementary Fig. 5). Similar trends were seen in the outcomes of the EORTC QLQ-C30 symptom scales, EORTC BM-22, HADS, and CIS-Fatigue (Supplementary Figs. 6–8). In patients who received 1–3 injections, clinically relevant and statistically significant deterioration was found in psychical, role, emotional and social functioning and in dyspnea, fatigue, and appetite (Supplementary Table 3). In patients who received 4-5 injections, clinically relevant and statistically significant HR-QoL deterioration was observed by end of treatment (Supplementary Table 4). When compared to baseline, fatigue, pain, dyspnea, appetite loss, constipation and diarrhea increased significantly during therapy in this subgroup. Furthermore, the outcomes of the HADS and CIS-Fatigue questionnaires revealed clinically relevant and statistically significant increase of psychological distress and fatigue over time in patients who discontinued radium-223 therapy. We did not find a statistically significant correlation between PSA response to radium-223 therapy and patterns in psychological distress (Supplementary Table 5).

### HR-QoL trajectory analysis

In patients with high EORTC QLQ-C30 summary scores at baseline, OS was significantly longer (median 14.2 months, 95% CI 12.8–15.7) than in patients with intermediate (median 12.4 months, 95% CI 9.5–15.3) and low baseline summary scores (median 7.7 months, 95% CI 3.4–11.9; *P* < 0.001; Supplementary Fig. 3B).

Based on changes in the summary scores over time, 50 (44%), 9 (8%), 10 (9%), 27 (24%), 9 (8%), and 9 (8%) patients were categorized as having deteriorated, stable low, stable intermediate, stable high, improved and fluctuating HR-QoL, respectively. In patients who discontinued therapy, HR-QoL deteriorated more frequently (58%) than in patients who received six radium-223 injections (37%). In addition, OS was significantly different among the HR-QoL classes (Supplementary Table 6).

When compared to patients who experienced HR-QoL deterioration (reference class), patients with baseline opioid use were more likely to have stable low HR-QoL over time (odds ratio 9.00, *P* < 0.05) and less likely to have a stable high HR-QoL over time (odds ratio 0.10, *P* < 0.05). Furthermore, patients in the stable high HR-QoL class were more likely to have high baseline hemoglobin and low ALP levels at baseline (Table 3).

**Table 3:** Trajectory analysis outcomes showing factors associated with class membership of health-related quality of life during radium-223 therapy.

	Class 1 Deteriorated (*N* = 50)	Class 2 Stable low (*N* = 9) OR (95% CI)		Class 3 Stable intermediate (*N* = 10) OR (95% CI)		Class 4 Stable high (*N* = 27) OR (95% CI)		Class 5 Improved (*N* = 8) OR (95% CI)		Class 6 Fluctuating (*N* = 9) OR (95% CI)	
Age	Reference	1.00	(0.92–1.09)	1.04	(0.96–1.13)	0.99	(0.94–1.05)	1.01	(0.93–1.10)	1.04	(0.96–1.14)
Partnership		0.66	(0.07–6.00)	*NE*	-	0.91	(0.25–3.36)	1.75	(0.30–10.27)	*NE*	-
Opioid use		9.00	(1.66–48.70)*	3.86	(0.94–15.76)	0.10	(0.01–0.80)*	3.21	(0.75–13.74)	0.74	(0.14–3.98)
Hemoglobin		1.34	(0.50–3.55)	1.22	(0.48–3.05)	2.29	(1.14–4.62)*	1.90	(0.68–5.29)	0.91	(0.36–2.31)
Alkaline phosphatase (log)		0.51	(0.04–7.54)	0.15	(0.01–2.83)	0.12	(0.02–0.90)*	3.72	(0.41–34.12)	0.12	(0.01–2.79)
Prostate-specific antigen (log)		0.90	(0.34–2.42)	0.55	(0.22–1.35)	1.29	(0.62–2.30)	3.05	(0.98–9.53)	1.11	(0.41–3.04)
Number of prior therapies		1.03	(0.51–2.08)	1.17	(0.60–2.26)	1.27	(0.80–2.00)	2.17	(1.12–4.22)*	0.92	(0.44–1.89)
ECOG performance status											
ECOG 1		2.89	(0.48–17.49)	10.11	(1.14–89.43)*	0.90	(0.34–2.44)	7.22	(0.78–67.14)	1.93	(0.38–9.67)
ECOG 2-3		6.50	(0.88–47.90)	8.67	(0.67–112.04)	0.27	(0.03–2.46)	13.00	(1.14–147.82)*	1.93	(0.38–9.67)

*CI* confidence interval, *ECOG* Eastern Cooperative Oncology Group, *NE* not evaluable due to low number of patients in class, *OR* odds ratio.

*P value < 0.05.

## Discussion

In this prospective cohort study, baseline cancer-specific and bone metastases-related HR-QoL, pain intensity, psychological distress and fatigue were worse in patients who discontinued radium-223 therapy. Additionally, patients who discontinued radium-223 therapy also experienced significant deterioration of HR-QoL, psychological distress and fatigue over time. In contrast, in patients who completed therapy stabilization of HR-QoL, psychological distress and fatigue were reached. Furthermore, descriptive trajectory analysis showed subgroups of patients with similar HR-QoL patterns over time. Baseline opioid use, hemoglobin, and ALP levels were identified as predictors of the different HR-QoL courses.

In this cohort of mCRPC patients, physical and role functioning were mostly affected HR-QoL domains. The identified impact on these functioning scales is in line with previously published patient-reported outcomes of Dutch mCRPC patients who were treated with various life-prolonging agents in daily practice, and reflects the pain and fatigue which mCRPC patients experience during daily activities [18]. Other studies in patients with less advanced mCRPC reported higher baseline scores on EORTC QLQ-C30 functioning domains [18, 34]. Hence, comparison of study outcomes is hampered by differences in administered therapies, disease stage of the patients, and the availability of other life-prolonging agents at the time of HR-QoL evaluation. Moreover, direct comparison with the HR-QoL outcomes of phase 3 studies is not possible due to the use of different HR-QoL instruments.

One previous study has evaluated HR-QoL by the EORTC QLQ-C30 in 30 mCRPC patients who underwent radium-223 therapy. This study reported lower HR-QoL scores and higher symptom burden at baseline when compared to our study. Notably, patients in that study were less pretreated with prior chemotherapy in only 37% of the patients. The authors found small worsening of patients’ role functioning after the first radium-223 injection, which stabilized during subsequent treatment cycles [35]. Furthermore, symptoms of dyspnea and diarrhea increased over time. However, the impact of this study was limited by the small cohort size and single-center study design.

It is known that mCRPC patients experience significant HR-QoL deterioration over time as a result of disease-related symptoms [5, 18]. We found significant deterioration in all EORTC functioning domains over time, except for cognitive functioning. The demonstrated increase in fatigue and dyspnea in the present study may be related to decreased hematologic function as a result of disease progression or adverse event of radium-223, whereas the increase in constipation scores may reflect a side effect of opioid use [9].

In the contemporary study, we showed that HR-QoL deterioration was mainly found in patients who discontinued radium-223 therapy. This finding underlines the importance of appropriate selection of patients for radium-223 therapy, in order to achieve the HR-QoL benefits of this therapy. Furthermore, since baseline HR-QoL score correlates with OS in our study and prior cohort studies, HR-QoL evaluation may be useful in clinical decision-making on treatment options in mCRPC [36, 37].

In the present cohort, we found that HR-QoL deterioration was accompanied by an increase in psychological distress and fatigue in patients who discontinued radium-223 therapy. Patients’ concerns about the discontinuation of radium-223 therapy and uncertainty about further systemic options for metastatic prostate cancer may explain the found increase of psychological distress. In a prior study including 63 mCRPC patients who were treated with radium-223, no significant variations in psychological status were detected, as measured by the EORTC QLQ-C30 and BM-22 questionnaires [38]. Although pain intensity significantly decreased throughout therapy, no association was found between psychological status and the observed pain relief. Importantly, specific instruments to assess psychological distress were not used in this study.

By performing trajectory analysis, we found that there is considerable variation in HR-QoL patterns during radium-223 therapy. Although 40% of the patients showed stable HR-QoL over time, HR-QoL deteriorated in most patients (44%). Opioid use, low hemoglobin, and high ALP levels at baseline were related to HR-QoL deterioration over time. These variables are known prognostic parameters that have been associated with the number of radium-223 injections and OS [39, 40]. These variables may be used to identify patients who need specific attention during follow-up, in order to preserve HR-QoL. However, due to the relatively small sample size of the trajectory classes, validation of our findings in larger cohorts is warranted.

The current study is limited by its observational nature. Decisions regarding radium-223 therapy were made by the local physicians without strict criteria for evaluation and (dis)continuation of therapy, reflecting the situation in daily clinical practice. Furthermore, we chose to evaluate patient-reported outcomes at three-time points. The interval of 12 weeks may have led to missing relevant information about patients’ HR-QoL status between the time points. However, increasing the frequency of HR-QoL assessments does increase patient burden and is likely to result in lower compliance rates [41]. The study is strengthened by the multicenter study design, the use of validated questionnaires and the high compliance rates at all time points, even in patients who discontinued therapy.

In conclusion, patients who completed radium-223 had better baseline HR-QoL and experienced stabilization of HR-QoL, psychological distress, and fatigue during treatment. Patients who used opioids and had low hemoglobin and high ALP levels at baseline were at higher risk of HR-QoL deterioration and therefore, specific attention during follow-up is indicated in these patients. The incorporation of HR-QoL evaluation in daily practice might help to treat physicians in clinical decision-making and the evaluation of treatment effects.

## Supplementary information

Supplementary material

## References

[1] Coleman RE (2006). Clinical features of metastatic bone disease and risk of skeletal morbidity. Clin Cancer Res.

[2] Saad F, Lipton A, Cook R, Chen YM, Smith M, Coleman R (2007). Pathologic fractures correlate with reduced survival in patients with malignant bone disease. Cancer.

[3] Fizazi K, Massard C, Smith M, Rader M, Brown J, Milecki P (2015). Bone-related parameters are the main prognostic factors for overall survival in men with bone metastases from castration-resistant prostate cancer. Eur Urol.

[4] Weinfurt KP, Li Y, Castel LD, Saad F, Timbie JW, Glendenning GA (2005). The significance of skeletal-related events for the health-related quality of life of patients with metastatic prostate cancer. Ann Oncol.

[5] Sullivan PW, Mulani PM, Fishman M, Sleep D (2007). Quality of life findings from a multicenter, multinational, observational study of patients with metastatic hormone-refractory prostate cancer. Qual Life Res.

[6] Nead KT, Sinha S, Yang DD, Nguyen PL (2017). Association of androgen deprivation therapy and depression in the treatment of prostate cancer: a systematic review and meta-analysis. Urol Oncol.

[7] Chipperfield K, Fletcher J, Millar J, Brooker J, Smith R, Frydenberg M (2013). Predictors of depression, anxiety and quality of life in patients with prostate cancer receiving androgen deprivation therapy. Psychooncology.

[8] Guan T, Santacroce SJ, Chen DG, Song L (2020). Illness uncertainty, coping, and quality of life among patients with prostate cancer. Psychooncology.

[9] Parker C, Nilsson S, Heinrich D, Helle SI, O’Sullivan JM, Fossa SD (2013). Alpha emitter radium-223 and survival in metastatic prostate cancer. N Engl J Med.

[10] Hoskin P, Sartor O, O’Sullivan JM, Johannessen DC, Helle SI, Logue J (2014). Efficacy and safety of radium-223 dichloride in patients with castration-resistant prostate cancer and symptomatic bone metastases, with or without previous docetaxel use: a prespecified subgroup analysis from the randomised, double-blind, phase 3 ALSYMPCA trial. Lancet Oncol.

[11] Parker C, Finkelstein SE, Michalski JM, O’Sullivan JM, Bruland O, Vogelzang NJ (2016). Efficacy and safety of radium-223 dichloride in symptomatic castration-resistant prostate cancer patients with or without baseline opioid use from the phase 3 ALSYMPCA trial. Eur Urol.

[12] Nilsson S, Cislo P, Sartor O, Vogelzang NJ, Coleman RE, O’Sullivan JM (2016). Patient-reported quality-of-life analysis of radium-223 dichloride from the phase III ALSYMPCA study. Ann Oncol.

[13] Nilsson S, Larsen RH, Fossa SD, Balteskard L, Borch KW, Westlin JE (2005). First clinical experience with alpha-emitting radium-223 in the treatment of skeletal metastases. Clin Cancer Res.

[14] Nilsson S, Strang P, Aksnes AK, Franzen L, Olivier P, Pecking A (2012). A randomized, dose-response, multicenter phase II study of radium-223 chloride for the palliation of painful bone metastases in patients with castration-resistant prostate cancer. Eur J Cancer.

[15] Badrising SK, Louhanepessy RD, van der Noort V, Kieffer J, Coenen J, Hamberg P (2022). Integrated analysis of pain, health-related quality of life, and analgesic use in patients with metastatic castration-resistant prostate cancer treated with Radium-223. Prostate Cancer Prostatic Dis.

[16] Parimi S, Bondy S, Tsang E, McKenzie MR, Bachand F, Aparicio M (2019). Pain response in a population-based study of radium-223 (Ra223) for metastatic castration-resistant prostate cancer. Can Urol Assoc J.

[17] Morris MJ, Sartor O, Vogelzang NJ, Shore ND, Cislo P, Bangerter K (2015). Effect of radium-223 dichloride (Ra-223) on pain from US EAP. J Clin Oncol.

[18] Kuppen MCP, Westgeest HM, van den Eertwegh AJM, Coenen J, van Moorselaar RJA, van den Berg P (2020). Health-related quality of life and pain in a real-world castration-resistant prostate cancer population: results from the PRO-CAPRI study in the Netherlands. Clin Genitourin Cancer.

[19] Nussbaum N, George DJ, Abernethy AP, Dolan CM, Oestreicher N, Flanders S (2016). Patient experience in the treatment of metastatic castration-resistant prostate cancer: state of the science. Prostate Cancer Prostatic Dis.

[20] Kretschmer A, Ploussard G, Heidegger I, Tsaur I, Borgmann H, Surcel C (2021). Health-related quality of life in patients with advanced prostate cancer: a systematic review. Eur Urol Focus.

[21] Scher HI, Morris MJ, Stadler WM, Higano C, Basch E, Fizazi K (2016). Trial design and objectives for castration-resistant prostate cancer: updated recommendations from the Prostate Cancer Clinical Trials Working Group 3. J Clin Oncol.

[22] Aaronson NK, Ahmedzai S, Bergman B, Bullinger M, Cull A, Duez NJ (1993). The European Organization for Research and Treatment of Cancer QLQ-C30: a quality-of-life instrument for use in international clinical trials in oncology. J Natl Cancer Inst.

[23] Chow E, Hird A, Velikova G, Johnson C, Dewolf L, Bezjak A (2009). The European Organisation for Research and Treatment of Cancer Quality of Life Questionnaire for patients with bone metastases: the EORTC QLQ-BM22. Eur J Cancer.

[24] Chow E, Nguyen J, Zhang L, Tseng LM, Hou MF, Fairchild A (2012). International field testing of the reliability and validity of the EORTC QLQ-BM22 module to assess health-related quality of life in patients with bone metastases. Cancer.

[25] Scott NW, Fayers P, Aaronson NK, Bottomley A, de Graeff A, Groenvold M, et al. EORTC QLQ-C30 reference values manual. *EORTC Quality of Life Group, Brussels, Belgium* 2008.

[26] Osoba D, Rodrigues G, Myles J, Zee B, Pater J (1998). Interpreting the significance of changes in health-related quality-of-life scores. J Clin Oncol.

[27] Cleeland CS (2006). The measurement of pain from metastatic bone disease: capturing the patient’s experience. Clin Cancer Res.

[28] Zigmond AS, Snaith RP (1983). The hospital anxiety and depression scale. Acta Psychiatr Scand.

[29] Puhan MA, Frey M, Büchi S, Schünemann HJ (2008). The minimal important difference of the hospital anxiety and depression scale in patients with chronic obstructive pulmonary disease. Health Qual Life Outcomes.

[30] Vercoulen JH, Swanink CM, Fennis JF, Galama JM, van der Meer JW, Bleijenberg G (1994). Dimensional assessment of chronic fatigue syndrome. J Psychosom Res.

[31] Worm-Smeitink M, Gielissen M, Bloot L, van Laarhoven HWM, van Engelen BGM, van Riel P (2017). The assessment of fatigue: psychometric qualities and norms for the Checklist individual strength. J Psychosom Res.

[32] Giesinger JM, Kieffer JM, Fayers PM, Groenvold M, Petersen MA, Scott NW (2016). Replication and validation of higher order models demonstrated that a summary score for the EORTC QLQ-C30 is robust. J Clin Epidemiol.

[33] Husson O, de Rooij BH, Kieffer J, Oerlemans S, Mols F, Aaronson NK (2020). The EORTC QLQ-C30 summary score as prognostic factor for survival of patients with cancer in the “Real-World”: results from the population-based PROFILES registry. Oncologist.

[34] Westdorp H, Creemers JHA, van Oort IM, Mehra N, Hins-de Bree SM, Figdor CG (2020). High health-related quality of life during dendritic cell vaccination therapy in patients with castration-resistant prostate cancer. Front Oncol.

[35] Sraieb M, Hirmas N, Conrad R, Marinova M, Essler M, Herrmann K (2020). Assessing the quality of life of patients with metastatic castration-resistant prostate cancer with bone metastases receiving [223Ra]RaCl2 therapy. Medicines.

[36] Sullivan PW, Nelson JB, Mulani PM, Sleep D (2006). Quality of life as a potential predictor for morbidity and mortality in patients with metastatic hormone-refractory prostate cancer. Qual Life Res.

[37] Frantellizzi V, De Feo MS, Di Rocco A, Pontico M, Pani A, Farcomeni A (2020). Baseline quality of life predicts overall survival in patients with mCRPC treated with (223)Ra-dichloride. Hell J Nucl Med.

[38] De Vincentis G, Frantellizzi V, Follacchio GA, Farcomeni A, Pani A, Samaritani R (2019). No evidence of association between psychological distress and pain relief in patients with bone metastases from castration-resistant prostate cancer treated with 223Radium. Eur J Cancer Care.

[39] van der Doelen MJ, Mehra N, Hermsen R, Janssen MJR, Gerritsen WR, van Oort IM (2019). Patient selection for radium-223 therapy in patients with bone metastatic castration-resistant prostate cancer: new recommendations and future perspectives. Clin Genitourin Cancer.

[40] van der Doelen MJ, Stockhaus A, Ma Y, Mehra N, Yachnin J, Gerritsen WR (2021). Early alkaline phosphatase dynamics as biomarker of survival in metastatic castration-resistant prostate cancer patients treated with radium-223. Eur J Nucl Med Mol Imaging.

[41] Wintner LM, Sztankay M, Aaronson N, Bottomley A, Giesinger JM, Groenvold M (2016). The use of EORTC measures in daily clinical practice—a synopsis of a newly developed manual. Eur J Cancer.

