# Supplementary material for: Health-related quality of life, psychological distress, and fatigue in metastatic castration-resistant prostate cancer patients treated with radium-223 therapy
Source: Prostate Cancer Prostatic Dis. 2022 Jul 8;26(1):142–50. doi: 10.1038/s41391-022-00569-8 (PMC10023564; doi:10.1038/s41391-022-00569-8)

SUPPLEMENTARY MATERIAL

Health-related quality of life, psychological distress and fatigue in metastatic castration-resistant prostate cancer patients treated with radium-223 therapy

Maarten J. van der Doelen et al.

Prostate Cancer and Prostatic Diseases

2022

SUPPLEMENTARY TABLES AND FIGURES

		Page
**Suppl Table 1:**	Compliance rates with EORTC QLQ-C30, EORTC BM-22, BPI-SF, HADS and CIS-Fatigue questionnaires.	4
**Suppl Table 2:**	Comparison of patient-reported outcomes on cancer-specific and bone metastases-related health-related quality of life, psychological distress and fatigue over time in patients receiving 6 radium-223 injections.	5-7
**Suppl Table 3:**	Comparison of patient-reported outcomes on cancer-specific and bone metastases-related health-related quality of life, psychological distress and fatigue over time in patients receiving 1-3 radium-223 injections.	8-9
**Suppl Table 4:**	Comparison of patient-reported outcomes on cancer-specific and bone metastases-related health-related quality of life, psychological distress and fatigue over time in patients receiving 4-5 radium-223 injections.	10-12
**Suppl Table 5:**	Correlation of psychological distress and PSA response/progression	13
**Suppl Table 6:**	Overall survival among health-related quality of life trajectory classes based on EORTC-QLQ-C30 summary scores over time.	14
**Suppl Figure 1:**	Study schedule showing treatment schedule and time points (T0, T1, T2) of the provided questionnaires. Supplementary figure 1A shows the schedule for patients who completed radium-223 therapy. In case of early discontinuation of therapy, the patient was asked to complete an end-of-therapy questionnaire (time point T1 or T2) four weeks after the last received injection (B and C, respectively).	15
**Suppl Figure 2:**	Consort diagram patient enrollment regarding HADS and CIS-Fatigue questionnaires.	16

Suppl Figure 3: Overall survival of the total cohort (A) and overall survival stratified based on EORTC QLQ-C30 summary score categories at baseline (B). 17

Suppl Figure 4: Heat map of pain locations in metastatic castration-resistant prostate cancer patients prior to radium-223 therapy initiation (N=122). The color grid indicates the percentage of individuals that reported pain in the specific area. Dark red represents the most frequently reported area of pain, whereas white represents the least frequently reported area of pain. 18

Suppl Figure 5: Mean difference in scores from baseline in cancer-specific health-related quality of life (EORTC QLQ-C30 functional scales and global health status), comparing time point T1 to T0 (N=114) and T2 to T0 (N=91). Bars indicate the mean difference with 95% confidence intervals. 19

Suppl Figure 6: Course of cancer-specific health-related quality of life (EORTC QLQ-C30 symptom scales) over time in patients treated with radium-223 for metastatic castration-resistant prostate cancer. Bars indicate mean scores with 95% confidence intervals. 20-21

Suppl Figure 7: Course of bone metastases-related health-related quality of life (EORTC BM-22 functional and symptom scales) over time in patients treated with radium-223 for metastatic castration-resistant prostate cancer. Bars indicate mean scores with 95% confidence intervals. 22

Suppl Figure 8: Course of psychological distress and fatigue (HADS and CIS-Fatigue) over time in patients treated with radium-223 for metastatic castration-resistant prostate cancer. The dotted line indicates cut-off scores for the HADS total score and CIS-Fatigue. 23

Supplementary table 1. Compliance rate with questionnaires over time

Supplementary table 1A. Compliance rate with questionnaires over time – total cohort

	EORTC QLQ-C30 and BM-22<br>N (%)	BPI-SF<br>N (%)	HADS and CIS-F<br>N (%)
**T0 (baseline)**	122 (100)	122 (100)	96 (100)
**T1 (after 3 injections)**[a]	114 (93)	-	89 (93)
**T2 (after 6 injections)**[a]	91 (75)	-	69 (72)

[a] In case of preterm discontinuation of therapy, the follow-up questionnaire was complete four weeks after end of therapy

Supplementary table 1B. Compliance rate with questionnaires over time – patients receiving 1-3 injections

	EORTC QLQ-C30 and BM-22<br>N (%)	BPI-SF<br>N (%)	HADS and CIS-F<br>N (%)
**T0 (baseline)**	22 (100)	22 (100)	19 (100)
**T1 (after 3 injections)**[a]	17 (77)	-	15 (79)

[a] In case of preterm discontinuation of therapy, the follow-up questionnaire was complete four weeks after end of therapy

Supplementary table 1C. Compliance rate with questionnaires over time – patients receiving 4-5 injections

	EORTC QLQ-C30 and BM-22<br>N (%)	BPI-SF<br>N (%)	HADS and CIS-F<br>N (%)
**T0 (baseline)**	23 (100)	23 (100)	18 (100)
**T1 (after 3 injections)**	21 (91)	-	16 (89)
**T2 (after 4-5 injections)**[a]	16 (70)	-	13 (72)

[a] In case of preterm discontinuation of therapy, the follow-up questionnaire was complete four weeks after end of therapy

Supplementary table 1D. Compliance rate with questionnaires over time – patients receiving 6 injections

	EORTC QLQ-C30 and BM-22<br>N (%)	BPI-SF<br>N (%)	HADS and CIS-F<br>N (%)
**T0 (baseline)**	77 (100)	77 (100)	59 (100)
**T1 (after 3 injections)**	76 (99)	-	58 (98)
**T2 (after 6 injections)**	75 (97)	-	56 (95)

Supplementary table 2A. Comparison of patient-reported outcomes on cancer-specific and bone metastases-related health-related quality of life over time in patients receiving 6 radium-223 injections

	Comparison time points	N	Time point T0 Mean (SD) score		Time point T1 Mean (SD) score		Time point T2 Mean (SD) score		Mean difference	CRC	P value
**QLQ-C30 functional scales[a]**											
Global health status	T0-T1	76	65.7	(16.6)	66.2	(18.2)	-	-	+0.5	None	0.801
	T0-T2	75	65.7	(16.7)	-	-	58.8	(22.5)	-6.9	Small	**0.014**
	T1-T2	75	-	-	66.4	(18.2)	58.8	(22.5)	-7.7	Small	**0.002**
Physical functioning	T0-T1	76	73.5	(22.1)	71.2	(22.6)	-	-	-2.3	None	0.150
	T0-T2	75	73.8	(22.1)	-	-	66.9	(25.4)	-6.8	Small	**0.004**
	T1-T2	75	-	-	71.9	(22.0)	66.9	(25.4)	-5.0	Small	**0.009**
Role functioning	T0-T1	76	66.2	(29.2)	66.0	(30.1)	-	-	-0.2	None	0.962
	T0-T2	75	66.2	(29.4)	-	-	59.1	(30.2)	-7.1	Small	**0.042**
	T1-T2	75	-	-	66.4	(30.1)	59.1	(30.2)	-7.3	Small	**0.024**
Emotional functioning	T0-T1	76	79.6	(20.3)	82.6	(16.4)	-	-	+3.0	None	0.165
	T0-T2	75	79.3	(20.3)	-	-	78.1	(18.6)	-1.2	None	0.584
	T1-T2	75	-	-	82.4	(16.5)	78.1	(18.6)	-4.3	None	**0.012**
Cognitive functioning	T0-T1	76	86.4	(17.2)	88.6	(15.2)	-	-	+2.2	None	0.167
	T0-T2	75	86.2	(17.2)	-	-	84.9	(15.3)	-1.3	None	0.495
	T1-T2	75	-	-	88.4	(15.2)	84.9	(15.3)	-3.6	None	**0.032**
Social functioning	T0-T1	76	81.1	(21.8)	81.8	(24.7)	-	-	+0.7	None	0.770
	T0-T2	75	80.9	(21.9)	-	-	78.4	(24.8)	-2.4	None	0.321
	T1-T2	75	-	-	82.2	(24.6)	78.4	(24.8)	-3.8	None	0.104
**QLQ-C30 symptom scales[b]**											
Fatigue	T0-T1	76	34.5	(21.8)	35.8	(22.3)	-	-	+1.3	None	0.548
	T0-T2	75	34.7	(21.9)	-	-	46.4	(27.1)	+11.7	**Moderate**	**<0.001**
	T1-T2	75	-	-	35.6	(22.3)	46.4	(27.1)	+10.8	**Moderate**	**<0.001**
Nausea/vomiting	T0-T1	76	5.7	(14.0)	11.0	(16.9)	-	-	+5.3	Small	**0.002**
	T0-T2	75	5.1	(13.1)	-	-	15.1	(20.9)	+10.0	**Moderate**	**<0.001**
	T1-T2	75	-	-	10.2	(15.7)	15.1	(20.9)	+4.9	None	**0.044**
Pain	T0-T1	76	34.2	(27.5)	32.2	(27.8)	-	-	-2.0	None	0.557
	T0-T2	75	33.8	(27.4)	-	-	34.0	(27.5)	+0.2	None	0.948
	T1-T2	75	-	-	31.6	(27.3)	34.0	(27.5)	+2.4	None	0.286
Dyspnoea	T0-T1	76	14.9	(25.2)	19.7	(29.9)	-	-	+4.8	None	**0.040**
	T0-T2	75	14.7	(25.3)	-	-	23.1	(27.4)	+8.4	Small	**0.011**
	T1-T2	75	-	-	19.1	(29.6)	23.1	(27.4)	+4.0	None	0.181

Insomnia	T0-T1	76	20.2	(25.6)	20.6	(26.6)	-	-	+0.4	None	0.880
	T0-T2	75	20.4	(25.6)	-	-	20.4	(27.9)	0.0	None	1.000
	T1-T2	75	-	-	20.9	(26.7)	20.4	(27.9)	-0.4	None	0.885
Appetite loss	T0-T1	76	12.3	(23.6)	19.3	(27.4)	-	-	+7.0	Small	**0.015**
	T0-T2	75	11.6	(22.9)	-	-	27.6	(33.9)	+16.0	**Moderate**	**<0.001**
	T1-T2	75	-	-	18.2	(25.9)	27.6	(33.9)	+9.3	Small	**0.002**
Constipation	T0-T1	76	11.8	(21.6)	11.8	(21.6)	-	-	0.0	None	1.000
	T0-T2	75	11.6	(21.6)	-	-	13.8	(23.3)	+2.2	None	0.402
	T1-T2	75	-	-	11.1	(20.7)	13.8	(23.3)	+2.7	None	0.203
Diarrhoea	T0-T1	76	10.1	(26.1)	13.6	(22.6)	-	-	+3.5	None	0.261
	T0-T2	75	9.8	(26.2)	-	-	15.1	(22.8)	+5.3	Small	0.103
	T1-T2	75	-	-	12.9	(21.8)	15.1	(22.8)	+2.2	None	0.479
Financial difficulties	T0-T1	75	7.6	(18.6)	8.4	(25.2)	-	-	+0.9	None	0.698
	T0-T2	74	7.7	(18.7)	-	-	7.7	(18.7)	0.0	None	1.000
	T1-T2	75	-	-	8.4	(25.2)	7.6	(18.6)	-0.9	None	0.718
**BM-22 symptom scales[b]**											
Painful sites	T0-T1	76	23.9	(15.6)	21.8	(19.0)	-	-	-2.2	None	0.241
	T0-T2	75	23.8	(15.7)	-	-	21.2	(17.0)	-2.6	None	0.239
	T1-T2	75	-	-	20.9	(17.5)	21.2	(17.0)	-0.4	None	0.846
Pain characteristics	T0-T1	76	27.0	(23.0)	22.5	(23.4)	-	-	-4.5	None	0.106
	T0-T2	75	26.7	(22.9)	-	-	23.7	(23.2)	-3.0	None	0.332
	T1-T2	75	-	-	22.2	(23.5)	23.7	(23.2)	+1.5	None	0.494
**BM-22 functional scales[a]**											
Functional interference	T0-T1	76	75.2	(20.1)	77.8	(20.5)	-	-	+2.5	None	0.241
	T0-T2	75	75.4	(20.2)	-	-	75.1	(20.9)	-0.3	None	0.239
	T1-T2	75	-	-	78.4	(19.8)	75.1	(20.9)	-3.3	None	0.846
Psychosocial aspects	T0-T1	76	61.9	(16.2)	64.3	(14.7)	-	-	+2.4	None	0.113
	T0-T2	75	61.6	(16.0)	-	-	58.4	(17.1)	-3.2	None	0.131
	T1-T2	75	-	-	64.1	(14.7)	58.4	(17.1)	-5.8	Small	**0.004**

Mean health-related quality of life scores range from 0 to 100.
[a] Functional and global scales: high scores indicate high level of functioning.
[b] Symptom scales: high scores indicate high symptom burden.

CRC, Clinically relevant change, defined as small (5-10 points), moderate (10-20 points), or large (>20 points).

Supplementary table 2B. Comparison of patient-reported outcomes on psychological distress and fatigue over time in patients receiving 6 radium-223 injections

	Comparison time points	N	Time point T0 Mean (SD) score	Time point T1 Mean (SD) score	Time point T2 Mean (SD) score	Mean difference	CRC	P value
**HADS**[a]								
Anxiety score	T0-T1	58	5.1 (4.1)	4.2 (3.7)	- -	-0.9	No	**0.018**
	T0-T2	56	5.0 (3.9)	- -	4.6 (3.5)	-0.5	No	0.275
	T1-T2	56	- -	4.1 (3.6)	4.6 (3.5)	+0.5	No	0.163
Depression score	T0-T1	58	4.8 (3.8)	4.5 (3.6)	- -	-0.3	No	0.443
	T0-T2	56	4.8 (3.8)	- -	5.5 (4.0)	+0.6	No	0.169
	T1-T2	56	- -	4.4 (3.4)	5.5 (4.0)	+1.1	No	**0.006**
Total score	T0-T1	58	9.9 (7.5)	8.7 (6.8)	- -	-1.2	No	0.077
	T0-T2	56	9.9 (7.4)	- -	10.0 (6.9)	+0.2	No	0.841
	T1-T2	56	- -	8.5 (6.4)	10.0 (6.9)	+1.6	No	**0.011**
**CIS Fatigue**[a]								
Fatigue severity subscale	T0-T1	58	32.8 (13.6)	31.9 (13.0)	- -	-0.9	-	0.506
	T0-T2	56	32.7 (13.2)	- -	35.1 (13.2)	+2.3	-	0.142
	T1-T2	56	- -	31.7 (13.2)	35.1 (13.2)	+3.3	-	**0.012**

[a] High scores indicate high symptom burden.

Supplementary table 3A. Comparison of patient-reported outcomes on cancer-specific and bone metastases-related health-related quality of life over time in patients receiving 1-3 radium-223 injections

	**Comparison time points**	**N**	**Time point T0** Mean (SD) score	**Time point T1** Mean (SD) score	**Mean difference**	**CRC**	**P value**
**QLQ-C30 functional scales[a]**							
Global health status	T0-T1	17	56.9 (30.9)	50.0 (24.8)	-6.9	Small	0.308
Physical functioning	T0-T1	17	63.1 (27.4)	45.1 (31.6)	-18.0	Moderate	**0.001**
Role functioning	T0-T1	17	62.7 (36.1)	31.4 (34.3)	-31.4	**Large**	**0.001**
Emotional functioning	T0-T1	17	80.4 (19.3)	67.2 (28.0)	-13.2	Moderate	**0.034**
Cognitive functioning	T0-T1	17	84.3 (23.9)	73.5 (26.4)	-10.8	Moderate	0.077
Social functioning	T0-T1	17	81.4 (30.0)	56.9 (34.4)	-24.5	**Large**	**<0.001**
**QLQ-C30 symptom scales[b]**							
Fatigue	T0-T1	17	38.6 (29.7)	58.2 (31.6)	+19.6	Moderate	**0.013**
Nausea/vomiting	T0-T1	17	7.8 (13.3)	12.7 (20.9)	+4.9	None	0.369
Pain	T0-T1	17	45.1 (36.7)	53.9 (28.0)	+8.8	Small	0.350
Dyspnoea	T0-T1	17	25.5 (30.1)	41.2 (38.2)	+15.7	Moderate	**0.088**
Insomnia	T0-T1	17	19.6 (23.7)	23.5 (28.3)	+3.9	None	0.543
Appetite loss	T0-T1	17	15.7 (31.4)	43.1 (42.1)	+27.5	**Large**	**0.006**
Constipation	T0-T1	17	11.8 (26.2)	21.6 (28.7)	+9.8	Small	0.056
Diarrhoea	T0-T1	17	5.9 (13.1)	11.8 (26.2)	+5.9	Small	0.332
Financial difficulties	T0-T1	17	3.9 (11.1)	3.9 (11.1)	0.0	None	1.000
**BM-22 symptom scales[b]**							
Painful sites	T0-T1	17	24.3 (17.6)	27.5 (19.1)	+3.1	None	0.527
Pain characteristics	T0-T1	17	32.0 (23.9)	34.6 (25.4)	+2.6	None	0.668
**BM-22 functional scales[a]**							
Functional interference	T0-T1	17	74.3 (24.0)	59.8 (24.7)	-14.5	Moderate	**0.020**
Psychosocial aspects	T0-T1	17	58.2 (23.4)	50.0 (24.6)	-8.2	Small	**0.038**

Mean health-related quality of life scores range from 0 to 100.
[a] Functional and global scales: high scores indicate high level of functioning.
[b] Symptom scales: high scores indicate high symptom burden.

CRC, Clinically relevant change, defined as small (5-10 points), moderate (10-20 points), or large (>20 points).

Supplementary table 3B. Comparison of patient-reported outcomes on psychological distress and fatigue over time in patients receiving 1-3 radium-223 injections

	Comparison time points	N	Time point T0 Mean (SD) score	Time point T1 Mean (SD) score	Mean difference	CRC	P value
**HADS**[a]							
Anxiety score	T0-T1	15	5.1 (4.0)	7.6 (5.5)	+2.5	**Yes**	**0.014**
Depression score	T0-T1	15	6.8 (6.0)	10.1 (5.9)	+3.3	**Yes**	**0.011**
Total score	T0-T1	15	11.9 (9.6)	17.7 (10.5)	+5.8	**Yes**	**0.007**
**CIS Fatigue**[a]							
Fatigue severity subscale	T0-T1	15	30.3 (16.5)	39.6 (16.0)	+9.3	-	**0.005**

[a] High scores indicate high symptom burden.

Supplementary table 4A. Comparison of patient-reported outcomes on cancer-specific and bone metastases-related health-related quality of life over time in patients receiving 4-5 radium-223 injections

	Comparison time points	N	Time point T0 Mean (SD) score		Time point T1 Mean (SD) score		Time point T2 Mean (SD) score		Mean difference	CRC	P value
**QLQ-C30 functional scales[a]**											
Global health status	T0-T1	21	58.7	(22.0)	64.1	(26.7)	-	-	+5.4	Small	0.390
	T0-T2	16	60.9	(18.7)	-	-	36.5	(21.9)	-24.5	**Large**	**0.001**
	T1-T2	16	-	-	57.3	(20.2)	36.5	(21.9)	-20.8	**Large**	**0.003**
Physical functioning	T0-T1	21	72.1	(22.4)	64.1	(26.7)	-	-	-7.9	Small	0.126
	T0-T2	16	72.1	(22.2)	-	-	53.8	(22.4)	-18.3	Moderate	**<0.001**
	T1-T2	16	-	-	67.1	(22.9)	53.8	(22.4)	-13.3	Moderate	**0.025**
Role functioning	T0-T1	21	60.3	(35.1)	54.8	(32.1)	-	-	-5.5	Small	0.527
	T0-T2	16	62.5	(30.7)	-	-	31.3	(21.8)	-31.3	**Large**	**0.001**
	T1-T2	16	-	-	59.4	(28.5)	31.3	(21.8)	-28.1	**Large**	**0.001**
Emotional functioning	T0-T1	21	72.6	(26.8)	69.4	(26.1)	-	-	-3.2	None	0.496
	T0-T2	16	69.8	(26.7)	-	-	56.8	(24.8)	-13.0	Moderate	**0.022**
	T1-T2	16	-	-	68.2	(25.9)	56.8	(24.8)	-11.5	Moderate	**0.024**
Cognitive functioning	T0-T1	21	75.4	(23.3)	77.0	(20.1)	-	-	+1.6	None	0.666
	T0-T2	16	75.0	(22.8)	-	-	66.7	(27.2)	-8.3	Small	0.072
	T1-T2	16	-	-	76.0	(20.2)	66.7	(27.2)	-9.4	Small	0.083
Social functioning	T0-T1	21	76.2	(27.7)	69.0	(29.0)	-	-	-7.1	None	0.225
	T0-T2	16	74.0	(29.2)	-	-	51.0	(27.5)	-22.9	**Large**	**0.019**
	T1-T2	16	-	-	66.7	(32.2)	51.0	(27.5)	-15.6	Moderate	**0.034**
**QLQ-C30 symptom scales[b]**											
Fatigue	T0-T1	21	34.4	(24.6)	47.1	(21.3)	-	-	+12.7	Moderate	**0.041**
	T0-T2	16	34.0	(23.1)	-	-	68.8	(18.2)	+34.7	**Large**	**<0.001**
	T1-T2	16	-	-	47.9	(21.0)	68.8	(18.2)	+20.8	**Large**	**<0.001**
Nausea/vomiting	T0-T1	21	14.3	(26.5)	15.9	(20.1)	-	-	+1.6	None	0.793
	T0-T2	16	11.5	(23.3)	-	-	21.9	(31.5)	+10.4	Moderate	0.361
	T1-T2	16	-	-	17.7	(21.5)	21.9	(31.5)	+4.2	None	0.572
Pain	T0-T1	21	38.9	(30.0)	34.1	(30.5)	-	-	-4.8	None	0.602
	T0-T2	16	36.5	(28.0)	-	-	57.3	(29.8)	+20.8	**Large**	**0.004**
	T1-T2	16	-	-	37.5	(28.9)	57.3	(29.8)	+19.8	Moderate	**0.023**
Dyspnoea	T0-T1	21	14.3	(22.5)	30.2	(29.6)	-	-	+15.9	Moderate	**0.047**
	T0-T2	16	12.5	(20.6)	-	-	41.7	(28.5)	+29.2	**Large**	**0.002**
	T1-T2	16	-	-	27.1	(25.0)	41.7	(28.5)	+14.6	Moderate	**0.029**

Insomnia	T0-T1	21	19.0	(22.5)	20.6	(22.3)	-	-	+1.6	None	0.789
	T0-T2	16	20.8	(24.0)	-	-	31.3	(35.4)	+10.4	Moderate	0.289
	T1-T2	16	-	-	20.8	(20.6)	31.3	(35.4)	+10.4	Moderate	0.206
Appetite loss	T0-T1	21	20.6	(34.1)	31.7	(26.8)	-	-	+11.1	Moderate	0.110
	T0-T2	16	20.8	(36.3)	-	-	54.2	(29.5)	+33.3	**Large**	**0.002**
	T1-T2	16	-	-	31.3	(28.5)	54.2	(29.5)	+22.9	**Large**	**0.001**
Constipation	T0-T1	21	4.8	(12.0)	12.7	(19.7)	-	-	+7.9	Small	**0.021**
	T0-T2	16	2.1	(8.3)	-	-	33.3	(34.4)	+31.3	**Large**	**0.001**
	T1-T2	16	-	-	12.5	(20.6)	33.3	(34.4)	+20.8	**Large**	**0.007**
Diarrhoea	T0-T1	21	6.3	(22.7)	20.6	(32.4)	-	-	+14.3	Moderate	**0.035**
	T0-T2	16	2.1	(8.3)	-	-	16.7	(27.2)	+14.6	Moderate	**0.048**
	T1-T2	16	-	-	16.7	(27.2)	16.7	(27.2)	0.0	None	1.000
Financial difficulties	T0-T1	21	7.9	(18.0)	4.8	(15.9)	-	-	-3.2	None	0.493
	T0-T2	16	10.4	(20.1)	-	-	8.3	(14.9)	-2.1	None	0.580
	T1-T2	16	-	-	6.3	(18.1)	8.3	(14.9)	+2.1	None	0.718
**BM-22 symptom scales[b]**											
Painful sites	T0-T1	21	22.9	(17.1)	23.5	(19.6)	-	-	+0.6	None	0.866
	T0-T2	16	21.7	(17.5)	-	-	35.8	(24.6)	+14.2	Moderate	**0.016**
	T1-T2	16	-	-	26.3	(21.3)	35.8	(24.6)	+9.6	Small	0.077
Pain characteristics	T0-T1	21	26.5	(24.0)	21.2	(20.2)	-	-	-5.3	Small	0.408
	T0-T2	16	21.5	(19.7)	-	-	34.7	(24.3)	+13.2	Moderate	0.064
	T1-T2	16	-	-	22.2	(18.1)	34.7	(24.3)	+12.5	Moderate	**0.011**
**BM-22 functional scales[a]**											
Functional interference	T0-T1	21	74.4	(20.1)	71.0	(21.4)	-	-	-3.4	None	0.466
	T0-T2	16	74.0	(20.8)	-	-	60.7	(25.8)	-13.3	Moderate	0.085
	T1-T2	16	-	-	70.3	(21.5)	60.7	(25.8)	-9.6	Small	0.077
Psychosocial aspects	T0-T1	21	58.3	(21.5)	52.9	(18.7)	-	-	-5.4	Small	0.208
	T0-T2	16	58.8	(22.4)	-	-	43.1	(23.1)	-15.8	Moderate	**0.006**
	T1-T2	16	-	-	55.2	(18.9)	43.1	(23.1)	-12.2	Moderate	**0.003**

Mean health-related quality of life scores range from 0 to 100.
[a] Functional and global scales: high scores indicate high level of functioning.
[b] Symptom scales: high scores indicate high symptom burden.

CRC, Clinically relevant change, defined as small (5-10 points), moderate (10-20 points), or large (>20 points).

Supplementary table 4B. Comparison of patient-reported outcomes on psychological distress and fatigue over time in patients receiving 4-5 radium-223 injections

	**Comparison time points**	**N**	**Time point T0** Mean (SD) score	**Time point T1** Mean (SD) score	**Time point T2** Mean (SD) score	**Mean difference**	**CRC**	**P value**
**HADS[a]**								
Anxiety score	T0-T1	16	6.6 (3.1)	6.9 (3.8)	- -	+0.3	No	0.736
	T0-T2	13	6.8 (3.0)	- -	7.7 (4.8)	+0.9	No	0.384
	T1-T2	13	- -	7.4 (3.5)	7.7 (4.8)	+0.3	No	0.743
Depression score	T0-T1	16	6.4 (3.8)	7.8 (3.9)	- -	+1.3	No	0.071
	T0-T2	13	6.5 (3.9)	- -	10.1 (5.0)	+3.5	**Yes**	**<0.001**
	T1-T2	13	- -	7.9 (3.8)	10.1 (5.0)	+2.2	**Yes**	**0.044**
Total score	T0-T1	16	13.1 (6.2)	14.6 (6.3)	- -	+1.6	No	0.229
	T0-T2	13	13.3 (6.0)	- -	17.8 (8.8)	+4.5	**Yes**	**0.016**
	T1-T2	13	- -	15.3 (5.9)	17.8 (8.8)	+2.5	No	0.174
**CIS Fatigue[a]**								
Fatigue severity subscale	T0-T1	16	36.6 (11.9)	40.1 (11.5)	- -	+3.6	-	0.129
	T0-T2	13	36.6 (8.9)	- -	44.8 (8.7)	+8.2	-	**0.009**
	T1-T2	13	- -	41.9 (8.6)	44.8 (8.7)	+2.9	-	0.214

[a] High scores indicate high symptom burden.

Supplementary table 5. Correlation of psychological distress and PSA response/progression

PSA progression was defined as a confirmed ≥25% increase in PSA blood levels (from baseline or nadir) during radium-223 therapy, according to Prostate Cancer Working Group 3 criteria (Scher et al, J Clin Oncol, 2016). A PSA response was defined as a 30% or greater reduction in PSA level during therapy, according to the ALSYMPCA trial definition. Based on the clinically relevant change of 3 points change for the total HADS score, changes in total HADS scores over time were classified as deteriorated, stable, improved and fluctuating.

Eighty-nine patients had completed at least two HADS questionnaires and in these patients trajectory analysis of psychological distress was possible. Data on the PSA course during radium-223 were available in all of these 89 patients. In this subgroup, 14 of 89 patients (15.7%) had a PSA response and 80 of 89 patients (89.9%) experienced PSA progression during radium-223 therapy. We did not find a statistically significant correlation between psychological distress and PSA response/progression. However, these analyses may be underpowered due to the low number of subjects in each HADS subgroup.

	PSA response	No PSA response	Total
**Deteriorated HADS**	3 (19%)	13 (81%)	16 (100%)
**Stable HADS**	2 (8%)	24 (92%)	26 (100%)
**Improved HADS**	6 (18%)	28 (82%)	34 (100%)
**Fluctuating HADS**	3 (23%)	10 (77%)	13 (100%)
**Total**	14 (16%)	75 (84%)	89 (100%)

P = 0.572 (Pearson Chi Square test)

	PSA progression	No PSA progression	Total
**Deteriorated HADS**	14 (88%)	2 (13%)	16 (100%)
**Stable HADS**	25 (96%)	1 (4%)	26 (100%)
**Improved HADS**	29 (85%)	5 (15%)	34 (100%)
**Fluctuating HADS**	12 (92%)	1 (8%)	13 (100%)
**Total**	80 (90%)	9 (10%)	89 (100%)

P = 0.553 (Pearson Chi Square test)

Supplementary table 6. Overall survival among health-related quality of life trajectory classes based on EORTC-QLQ-C30 summary scores over time

		N (%)	Median OS (months)	95% CI	*P* value
**Class based on EORTC-QLQ-C30 summary scores over time**					
1	Deteriorated HR-QoL	50 (43.9)	10.9	7.3-14.5	**<0.001**
2	Stable low HR-QoL	9 (7.9)	8.5	6.0-11.1	
3	Stable intermediate HR-QoL	10 (8.8)	12.9	7.3-18.4	
4	Stable high HR-QoL	27 (23.7)	25.6	11.4-39.8	
5	Improved HR-QoL	9 (7.9)	16.7	11.2-22.2	
6	Fluctuating HR-QoL	9 (7.9)	13.9	7.7-20.2	
**Total cohort**		114 (100)	13.2	11.6-14.8	-

CI, confidence interval; EORTC, European Organization for Research and Treatment of Cancer; HR-QoL, health-related quality of life; OS, overall survival.

Suppl Figure 1

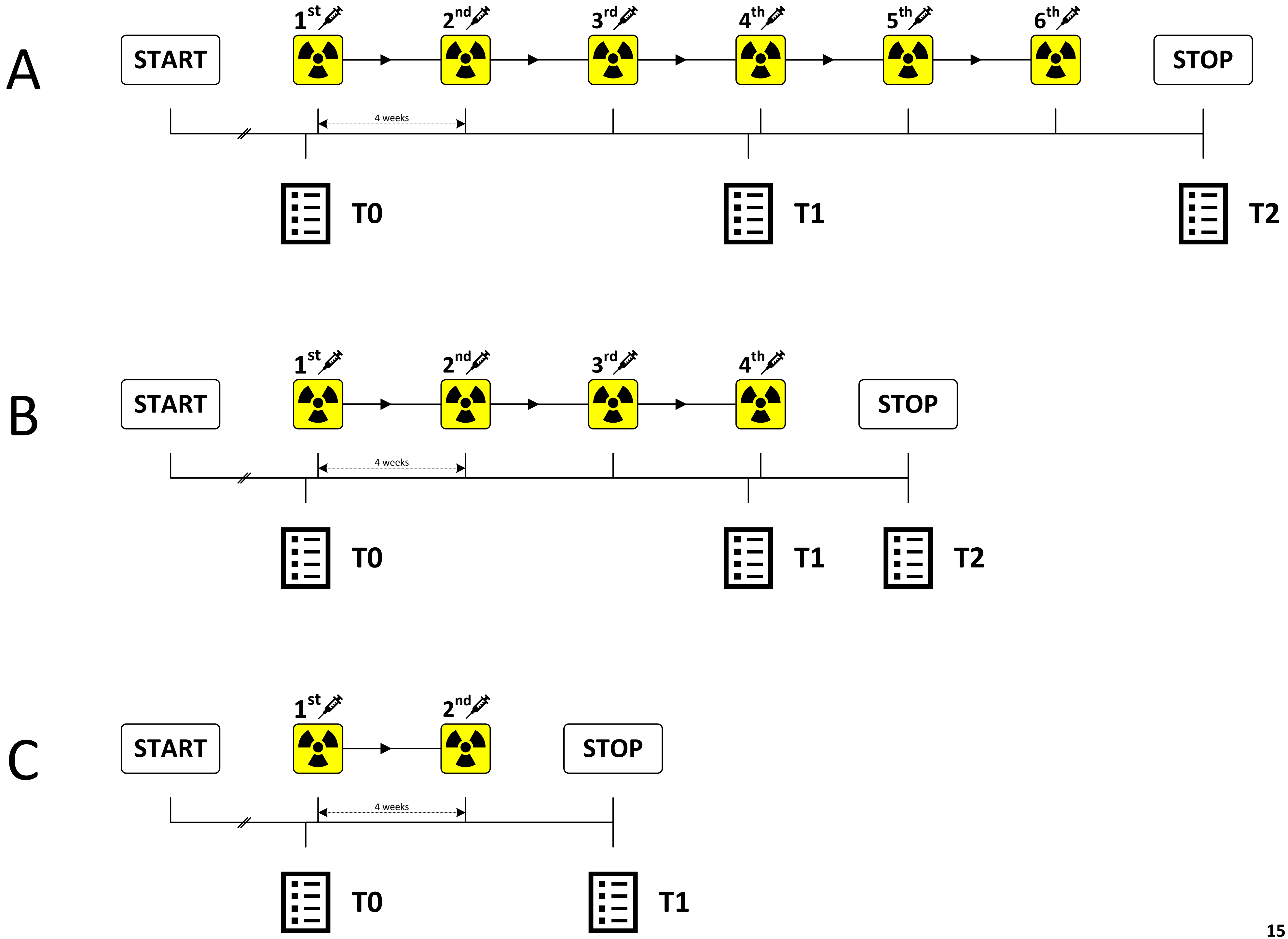

Suppl Figure 2

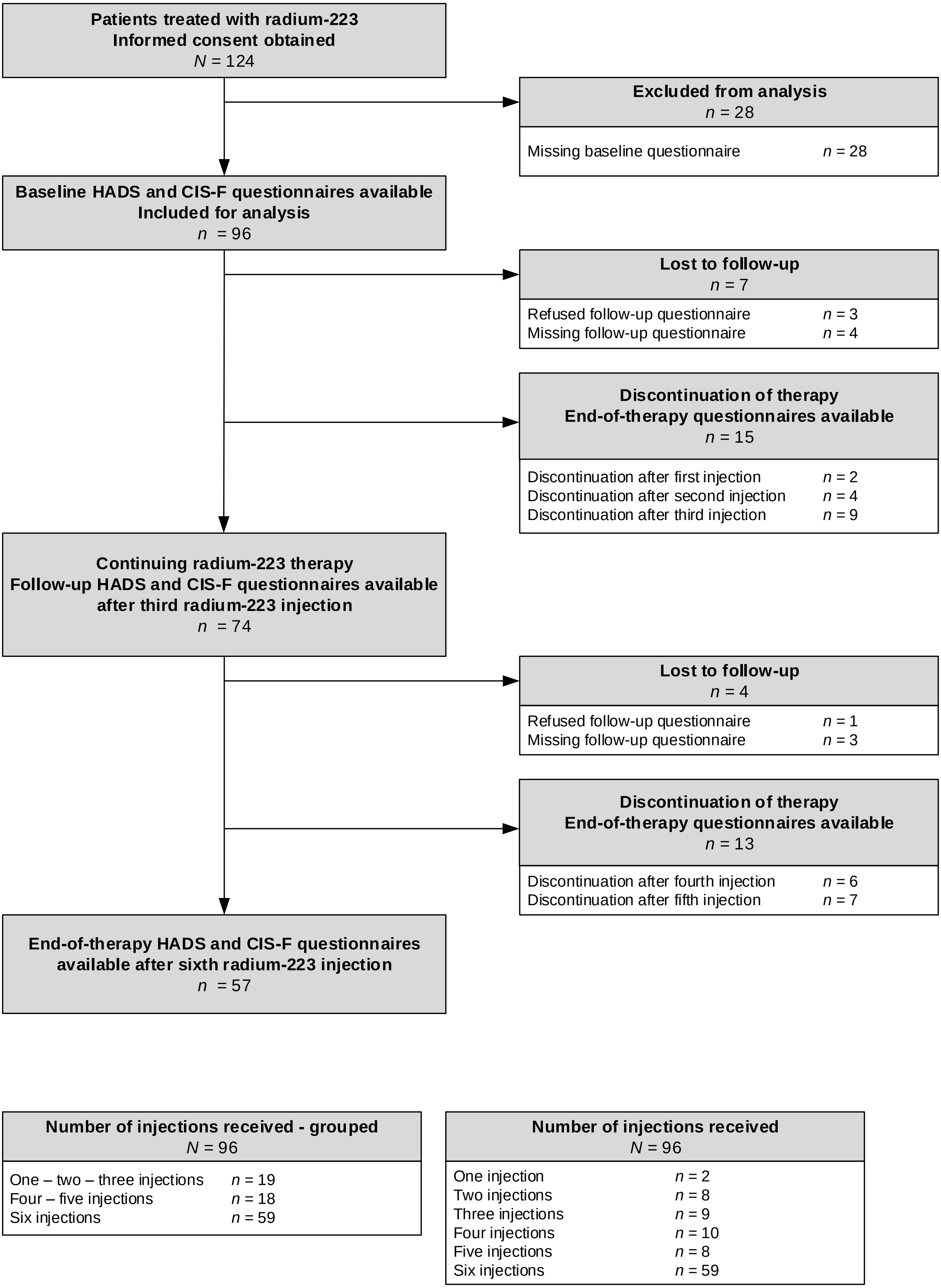

Suppl Figure 3

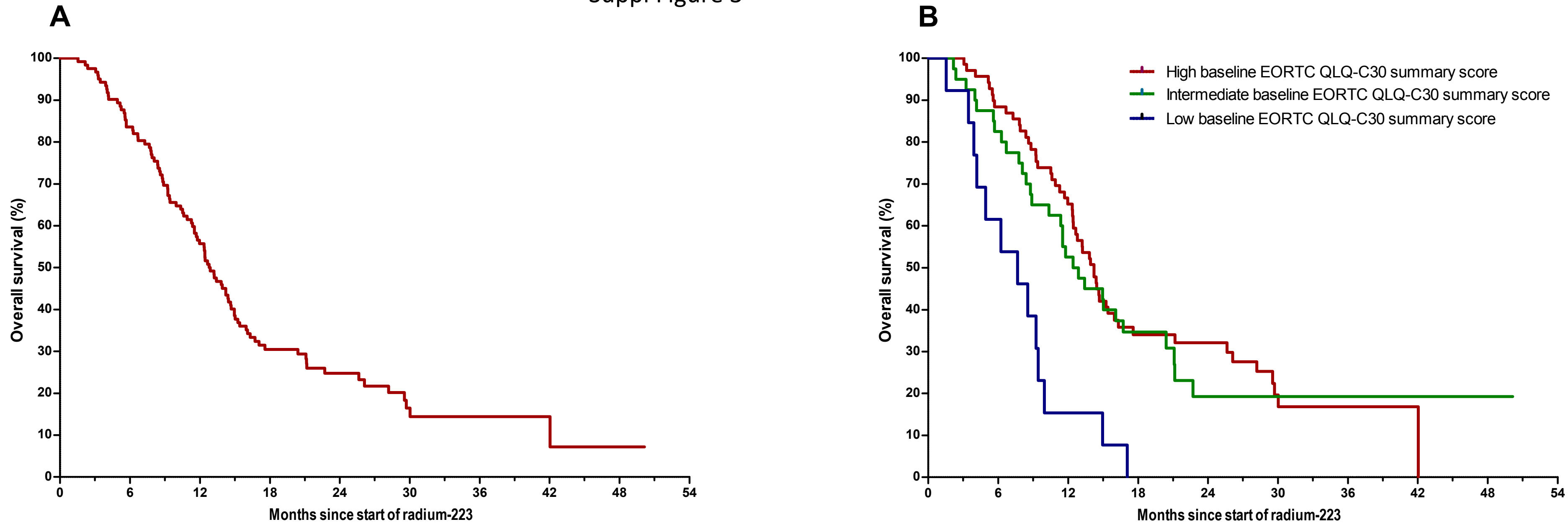

Patients at risk (A)

	0	6	12	18	24	30	36	42	48	54
Cohort	122	102	68	32	21	8	3	2	1	0

Patients at risk (B)

	0	6	12	18	24	30	36	42	48	54
High	69	61	45	19	16	7	2	1	0	0
Intermed	40	33	21	13	5	1	1	1	1	0
Low	13	8	2	0	0	0	0	0	0	0

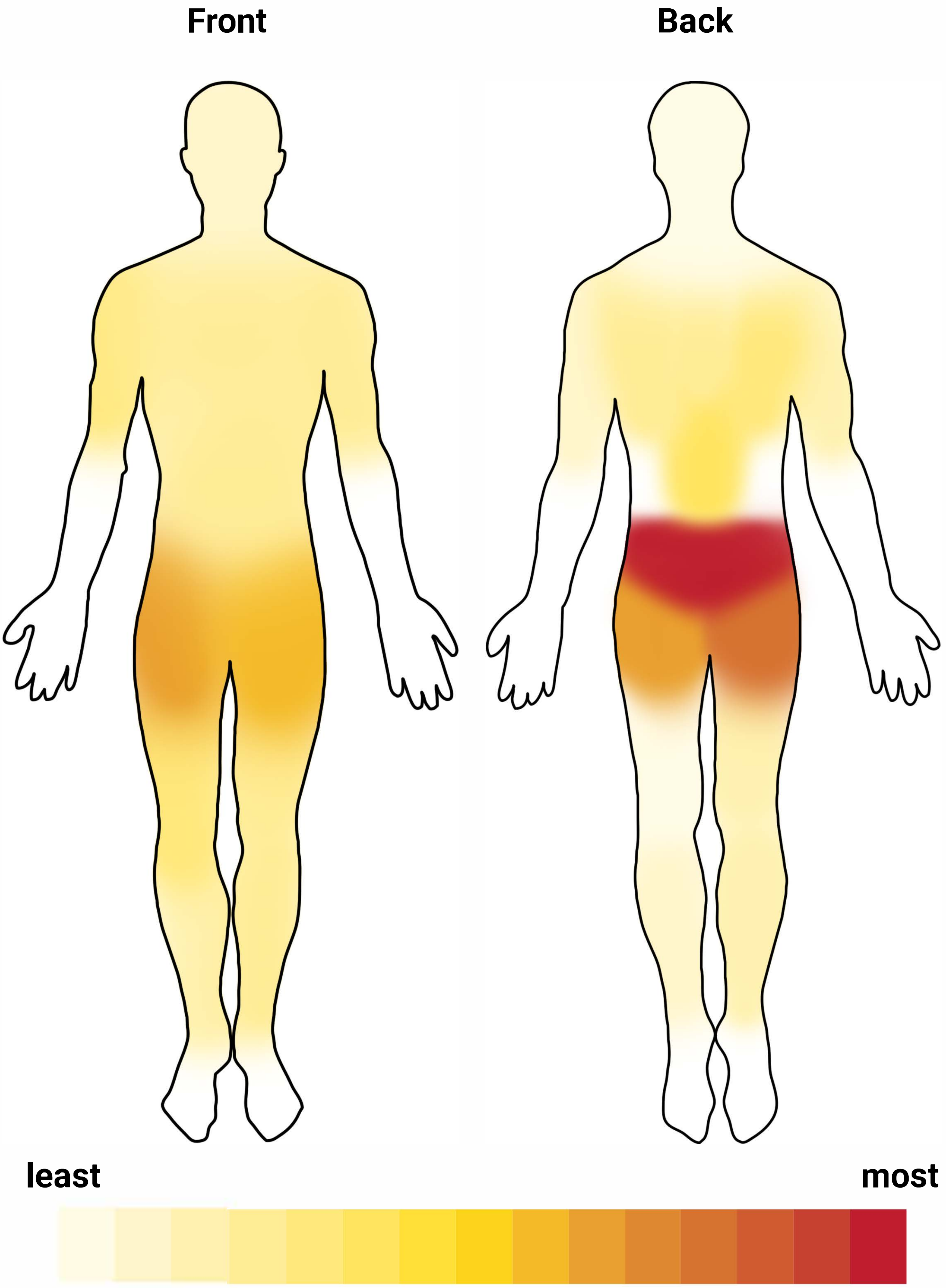
Front
Back
least
most

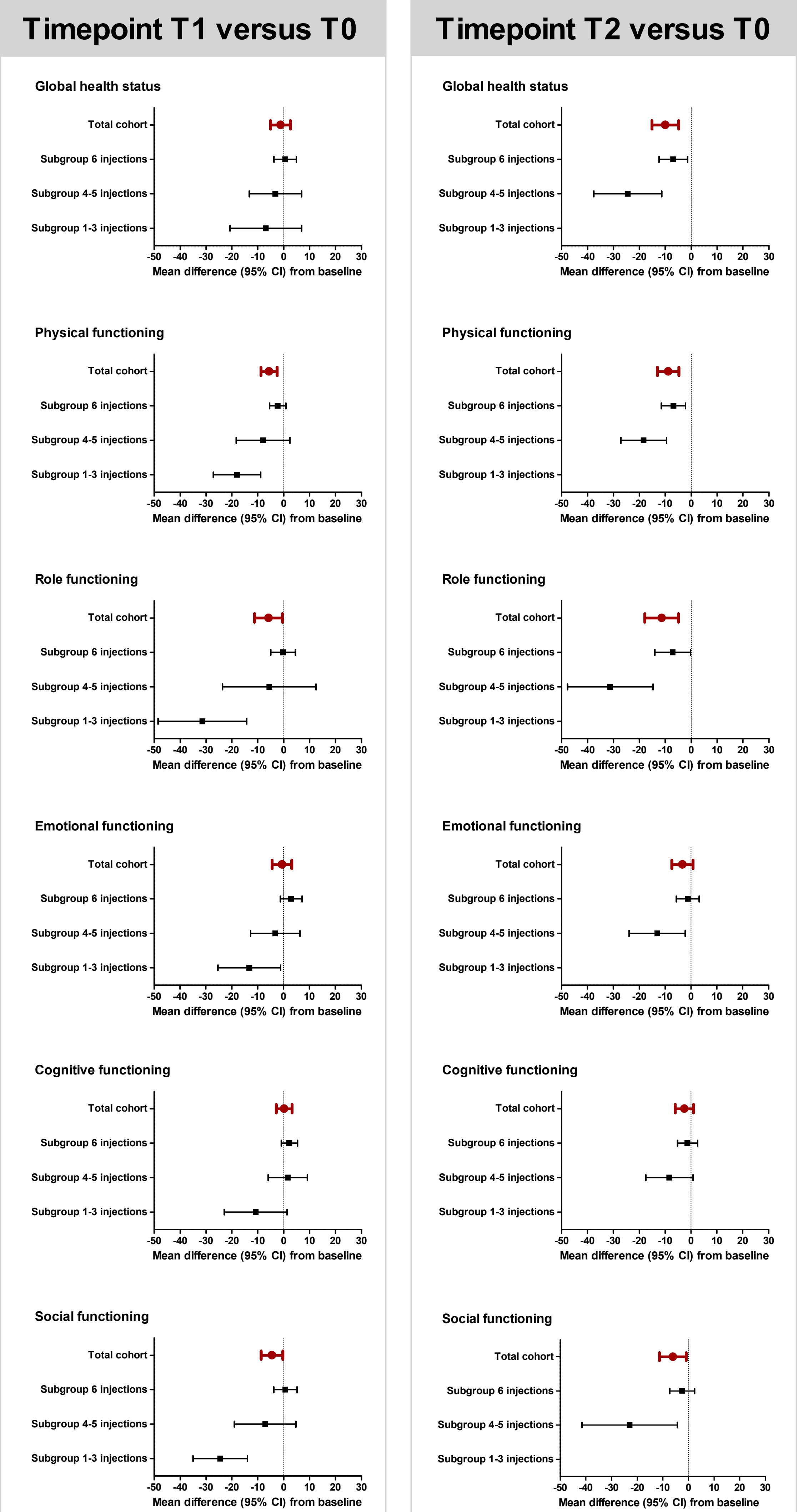

Suppl Figure 5

Suppl Figure 6

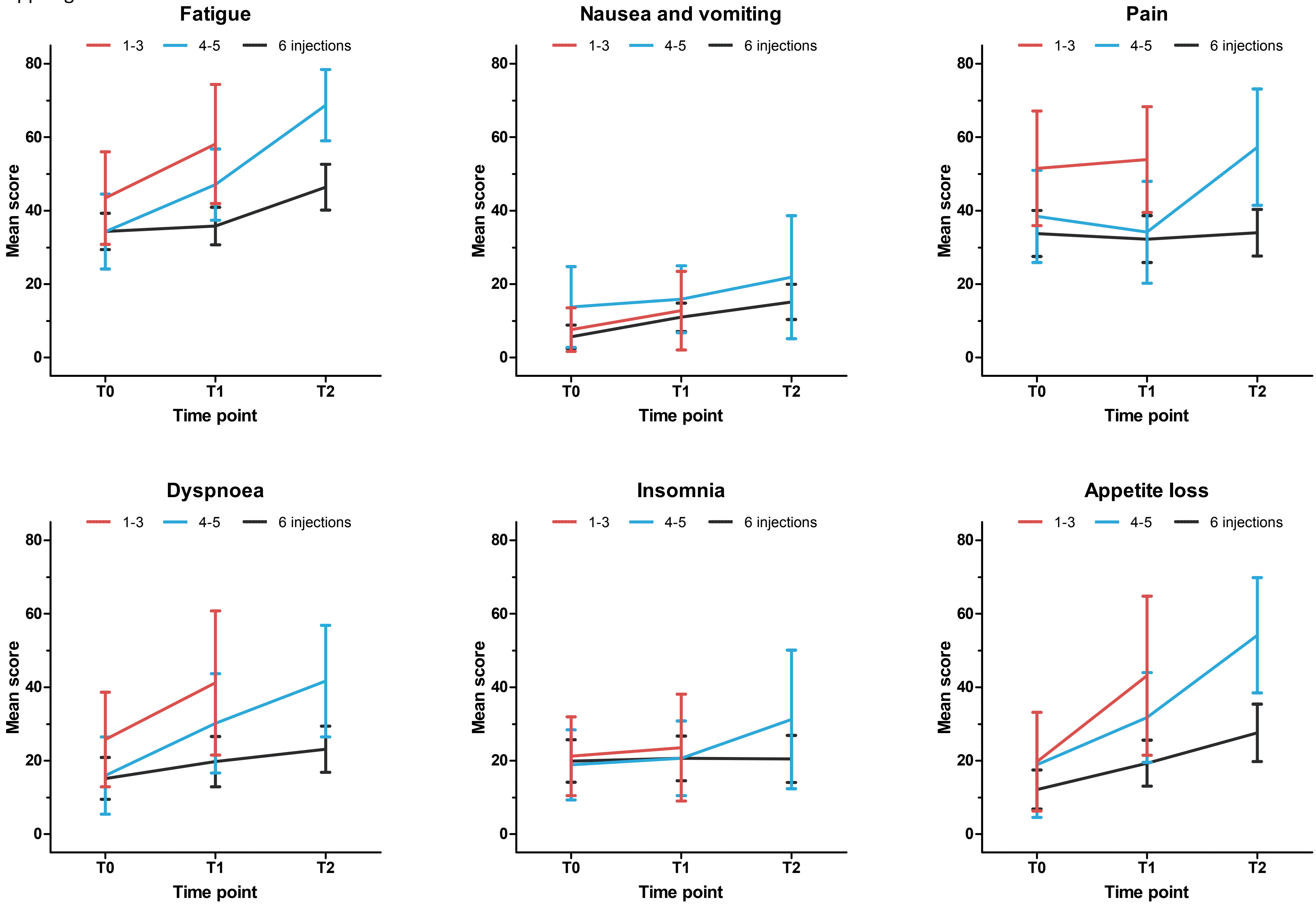

Suppl Figure 6

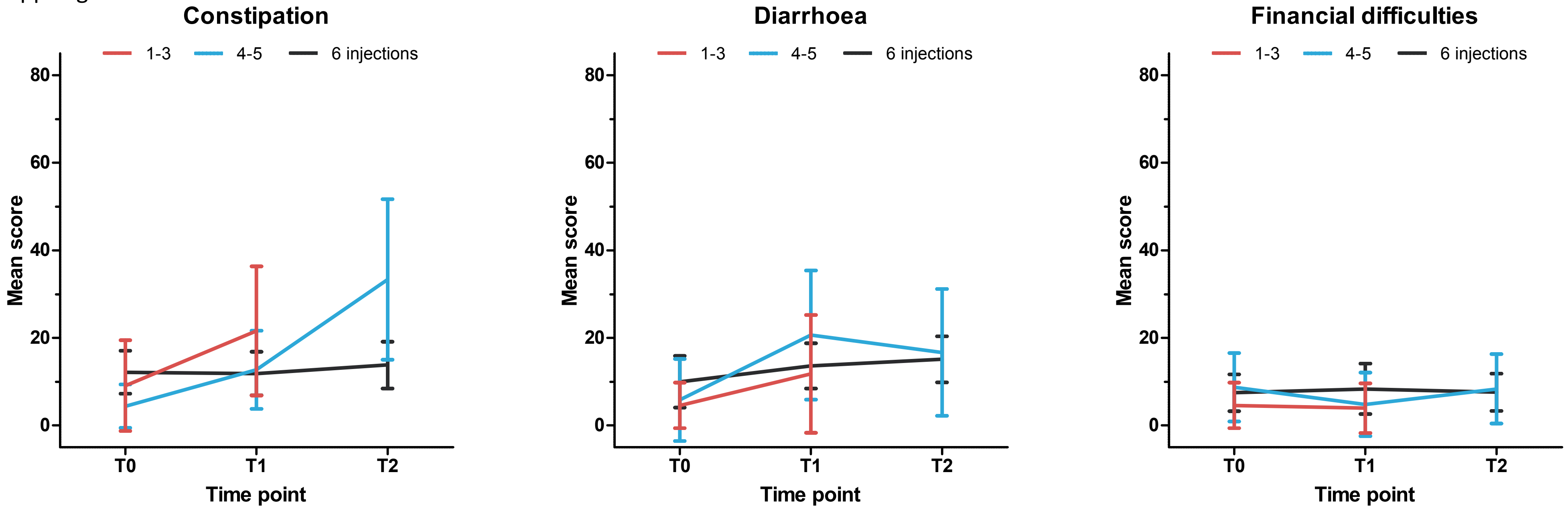

Suppl Figure 7

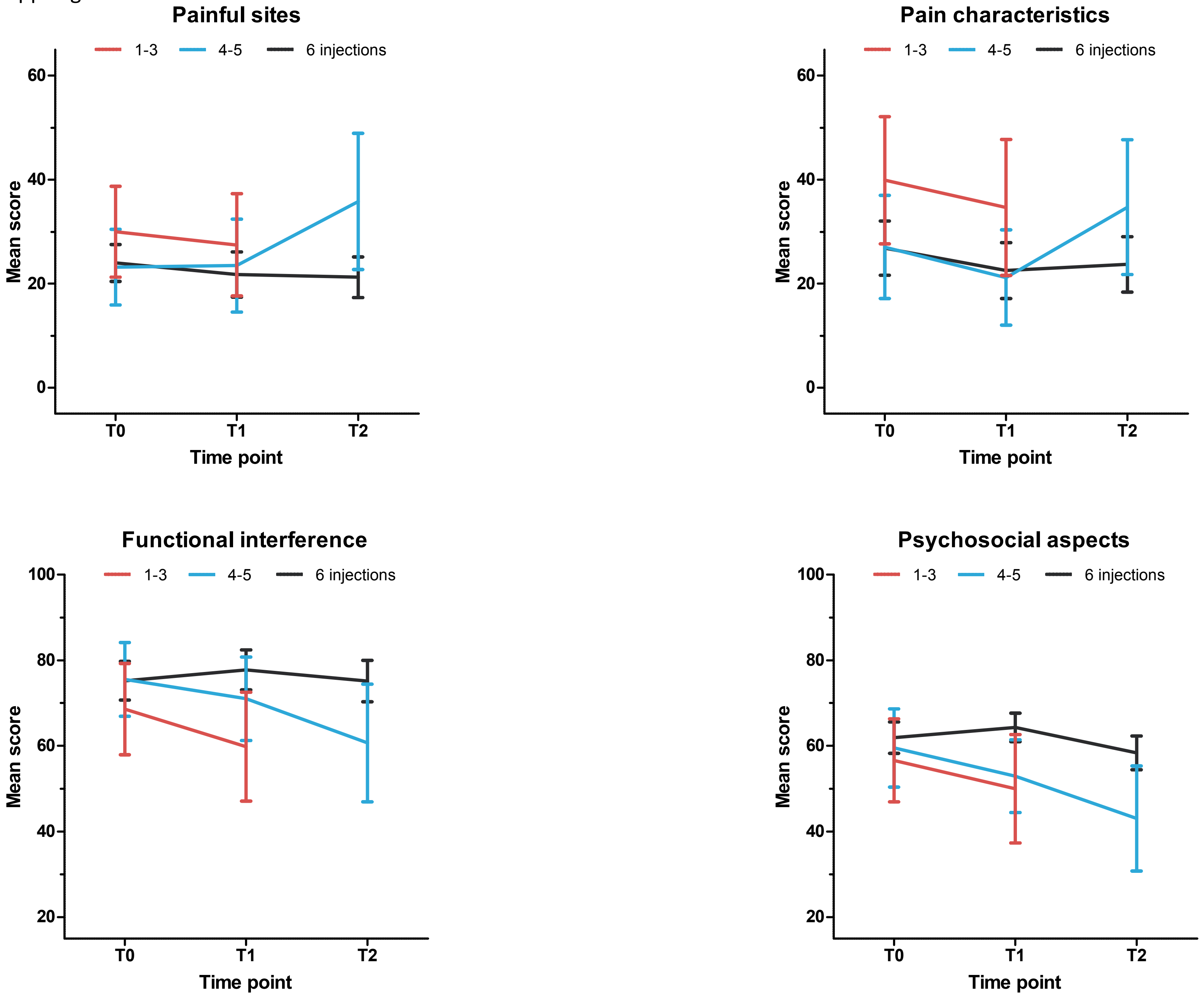

Suppl Figure 8

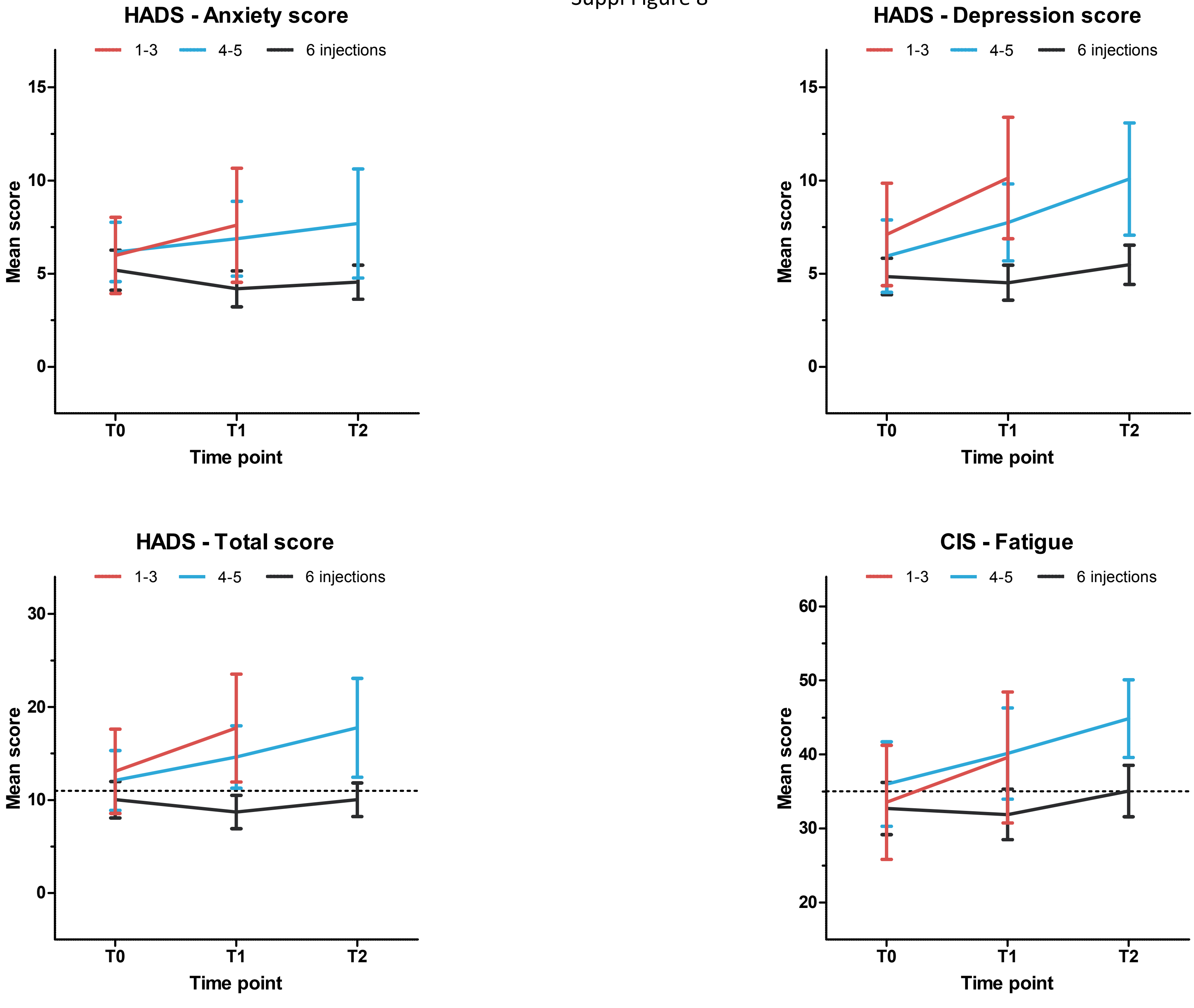

Supplement: Supplementary file 1 — Supplementary material [file 41391_2022_569_MOESM1_ESM.pdf]
